# In-hospital mortality of heat-related disease associated with wet bulb globe temperature: a Japanese nationwide inpatient data analysis

**DOI:** 10.1007/s00484-025-02867-x

**Published:** 2025-03-04

**Authors:** Kazuha Nakamura, Akira Okada, Hideaki Watanabe, Kazutaka Oka, Yasushi Honda, Hiroki Matsui, Kiyohide Fushimi, Hideo Yasunaga, Yoonhee Kim

**Affiliations:** 1 Department of Clinical Epidemiology and Health Economics, School of Public Health, The University of Tokyo, Tokyo, Japan; 2 Division of Emergency and Transport Services, National Center for Child Health and Development, Tokyo, Japan; 3 Department of Prevention of Diabetes and Lifestyle-Related Diseases, Graduate School of Medicine, The University of Tokyo, Tokyo, Japan; 4 Center for Climate Change Adaptation, National Institute for Environmental Studies, Ibaraki, Japan; 5 Department of Health Services Research, Graduate School of Medicine, The University of Tokyo, Tokyo, Japan; 6 Department of Health Policy and Informatics, Graduate School of Medicine, Tokyo Medical and Dental University, Tokyo, Japan; 7 Department of Global Environmental Health, Graduate School of Medicine, The University of Tokyo, 7-3-1 Hongo, Bunkyo-ku, Tokyo, 113-0033 Japan

**Keywords:** Heatstroke, Hospital mortality, Inpatient, WBGT

## Abstract

Heat-related diseases have become a significant public health concern. Studies have shown that susceptibility to heat varies among regions; however, most studies used aggregated data on emergency transport in the regions. The present study used a nationwide inpatient database in Japan and examined the association between regional differences in Wet Bulb Globe Temperature (WBGT) and in-hospital mortality in patients with a heat-related disease, with adjustment for individual-level characteristics. We retrospectively identified participants from the Japanese Diagnosis Procedure Combination inpatient database during the five warmest months of the year (May 1 to September 30) from 2011 to 2019. We calculated the long-term average daily maximum WBGT for the prefectures and categorized the prefectures into three areas (low-, middle-, and high-WBGT). We conduced multivariable logistic regression analyses to compare in-hospital mortality between the WBGT areas, adjusting for individual-level covariates (including age, sex, body mass index, and comorbidities). A total of 82,250 patients were admitted for heat-related diseases. The mean age was 63.2 (standard deviation, 25.0) years, and 63.7% were male. In the multivariable logistic regression analysis, the low-WBGT area had a higher in-hospital mortality than that had by the high-WBGT area (odds ratio, 1.32; 95% confidence interval, 1.15–1.52), whereas no significant difference was observed between the middle- and high-WBGT areas (odds ratio, 1.00; 95% confidence interval, 0.89–1.12). After adjusting for individual-level risk factors, in-hospital death was more likely to occur in patients with heat-related diseases in lower WBGT areas compared with those in higher WBGT areas.

**Supplementary Information:**

The online version contains supplementary material available at 10.1007/s00484-025-02867-x.

## Introduction

Heat-related diseases can potentially lead to death caused by multiple organ failure and have become an important public health issue as global warming progresses (Mora et al. 2017; Sorensen and Hess 2022). Risk factors for heat-related diseases include personal characteristics, such as advanced age, obesity, medication use, comorbidities, and environmental factors, such as unusual heatwave and the local climate (Westwood et al. 2021; Roberts et al. 2023). The accumulation of evidence on heat-related diseases has increasingly become important for implementing effective public health measures against heat-related diseases.

In recent years, research using diverse metrics to evaluate the impact of extreme heat on mortality and morbidity has steadily increased. These studies have utilized a wide range of heat stress indicators, from ambient temperature to composite indices such as the Wet-Bulb Globe Temperature (WBGT), apparent temperature, predicted mean vote, physiologically equivalent temperature, and the universal thermal climate index (Di Napoli et al. 2019; Spangler et al. 2022). Among these indices, the WBGT, which incorporates measurements of natural wet-bulb temperature, globe temperature, and dry-bulb temperature (Yaglou and Minard 1957), was adopted in 2021 as the primary heat exposure metric in Japan’s Heat Health Warning System. The WBGT is widely regarded as one of the most reliable heat stress indices in Japan (Guo et al. 2024).

Previous studies have also suggested that the alarming threshold for preventing the development of heat-related diseases should be based on the vulnerability of the local population (for example, heat tolerance varies by different levels of heat acclimatization). Therefore, public health policies against heat-related diseases should consider national-level policies, as well as the characteristics of the local population, which could contribute to improving heat health warning systems tailored to the local climate.

Japan’s climate extends from subtropical to subarctic, and several studies have analyzed regional differences in heat-related disease incidence in Japan from a meteorological perspective, such as temperature or WBGT differences (Miyatake et al. 2012; Otani et al. 2021; Iwamoto and Ohashi 2021; Oka and Hijioka 2021; Ueno et al. 2021; Oka et al. 2022, 2023). Previous studies have reported that under the same heat exposure, susceptibility to heat may be higher in northern Japan than in southern Japan. However, these studies utilized aggregated data on emergency transport and did not focus on individual patient risk factors other than age. Therefore, assessing the severity of heat-related diseases across different regions by adjusting for various individual risk factors is essential. Additionally, it is crucial to focus on evaluating the individual risk factors that contribute to the mortality of heat-related diseases.

We aimed to explore the association between long-term prefectural level average daily maximum WBGT and in-hospital mortality due to heat-related diseases in Japan using a nationwide inpatient database linked with meteorological data, adjusting for individual risk factors.

## Methods

### Study design and population

This nationwide retrospective observational study adopted a cross-sectional design and collected data of patients hospitalized for heat-related diseases from the Diagnosis Procedure Combination database, a nationwide inpatient database in Japan. The database collects administrative claims data from more than 1,200 acute care hospitals, which accounts for approximately 90% of the tertiary emergency hospitals in Japan. This database includes data on patients’ age, sex, body height, body weight, smoking history, Japan Coma Scale score at admission, diagnoses (primary diagnosis and comorbidities), medical procedures performed, discharge status, and hospitalization cost. We retrospectively extracted the data of patients admitted with a main or admission-precipitating diagnosis of heat-related disease (International Classification of Diseases 10th Revision [ICD-10] codes: T67) and were admitted to the hospital from May 1 to September 30 and from 2011 to 2019. We excluded patients who had missing information on (i) age, (ii) Japan Coma Scale score, (iii) fiscal year of admission, or (iv) daily maximum WBGT at admission. We did not include the data from fiscal year 2020 to 2022 because the substantial impacts of the coronavirus disease 2019 on medical care and lifestyle could have affected the results. A validation study of the records in the database showed that the sensitivity and specificity of the recorded diagnoses were 50–80% and > 96%, respectively, while the specificity and sensitivity of the recorded procedures exceeded 90% (Yamana et al. 2017). The requirement for informed consent was waived because the dataset was de-identified.

### Wet bulb globe temperature

We employed WBGT from the Heat Stroke Prevention System provided by the Ministry of Environment Japan (MEOJ accessed on May 21 2024) as an index of heat exposure. In this system, the WBGT values were calculated using the following equation:

$$ \begin{array}{l}{\rm{WBGT}}\,{\rm{ = }}\,0.735 \times Ta + 0.0374 \times RH + 0.00292 \times Ta \times RH\\+ 7.619 \times SR - 4.557 \times S{R^2} - 0.0572 \times WS - 4.064\end{array} $$

where *Ta* is temperature (°C), *RH* is relative humidity (%), *SR* is solar radiation (kW/m^2^), and *WS* is wind speed (m/s).

This system provides actual observed WBGT values for 11 prefectural capitals. For other prefectural capitals, due to a lack of observed data for certain components, WBGT values are estimated using forecast data from the Japan Meteorological Agency as an alternative.

WBGT readings for each prefectural capital represented the WBGT for the respective prefectures.

We defined the three WBGT metrics for our analysis: (i) **Long-term local WBGT** was defined as the long-term average of daily maximum WBGT for each prefecture during the study period (the five warmest months of the year, from May 1 to September 30, between 2011 and 2019); (ii) **Local WBGT areas** were defined based on long-term local WBGT values, categorized into three groups using the first and third quartiles: low-WBGT area (20.8–25.2 °C), middle-WBGT area (25.2–26.4 °C), and high-WBGT area (26.4–29.3 °C); and (iii) **Short-term local WBGT** represented the daily maximum WBGT for each prefecture on the day of hospital admission. Individual patients were linked to their respective prefectures based on the location of the hospital where they were admitted.

### Outcomes of interest

The study outcome was all-cause in-hospital mortality.

### Covariates

We extracted data on age, sex, smoking history, body mass index (BMI) at admission, Japan Coma Scale score at admission, Charlson Comorbidity Index (CCI; a tool for predicting mortality by classifying or weighting comorbid conditions), comorbidities (myocardial infarction, congestive heart failure, peripheral vascular disease, cerebrovascular disease, dementia, diabetes, kidney disease, or mental disorder), complications (acute liver failure, acute renal injury, and disseminated intravascular coagulation), ambulance use, intensive care unit and high care unit admissions, treatments (mechanical ventilation, kidney replacement therapy, and catecholamine administration), fiscal year, month of admission, length of stay, and costs of hospitalization. BMI was categorized based on the World Health Organization (WHO) classification as follows: underweight (< 18.5 kg/m^2^), normal (18.5–24.9 kg/m^2^), overweight (25.0–29.9 kg/m^2^), and obese (≥ 30.0 kg/m^2^). Cases without BMI data were categorized as missing. The level of consciousness on admission was categorized based on the Japan Coma Scale score as follows: 0, alert consciousness; 1–3, awake without any stimuli; 10–30, aroused by some stimuli; and 100–300, coma. The Japan Coma Scale and Glasgow Coma Scale scores are well correlated (Nakajima et al. 2022). The comorbidities included CCI and mental disorder (F04, F05, F06, F07, F09, F1, F2, F3, F4, F5, F6, F7, F8, and F9) items, and their ICD-10 codes were selected based on previous studies (Quan et al. 2005). Laboratory data are not included in the database. Therefore, complications were defined using ICD-10 codes (acute liver failure, K72.0, K72.9, K75.9, K76.2, K76.8, K76.9, R16.0, R16.2, R74.0, R17 or Z94.4; acute kidney injury, K17; and disseminated intravascular coagulation, D65, D68.9, or D69.9), which were selected according to previous studies (Forns et al. 2019; Ohbe et al. 2019; Logan et al. 2020). Hospitalization costs recorded in Japanese yen were converted to US dollars (149 yen = $1).

### Statistical analysis

Baseline characteristics of the patients were described and compared among the three local WBGT areas. The averages or medians of the continuous variables were compared using analysis of variance or the Kruskal-Wallis test, and the proportions of categorical variables were compared using the chi-square test.

Multivariable logistic regression analyses were performed to estimate the odds ratios (ORs) and 95% confidence intervals (CIs) for in-hospital mortality across the three local WBGT areas. The model was adjusted for the following patient-level variables as covariates: age, sex, BMI at admission, smoking history, CCI, mental disorder, ambulance use, fiscal year, and month of admission. These covariates were selected according to previous literature and clinical relevance (Bouchama et al. 2007; Schmeltz and Gamble 2017; Gifford et al. 2019; Xu et al. 2019; Shimazaki et al. 2020; Marx et al. 2021; Osborne et al. 2023). Age and CCI were used as a continuous variable.

### Subgroup analyses

Subgroup analyses were conducted based on age, sex, BMI, and CCI. We set the age threshold at 75 years. In the BMI-based subgroup analysis, we excluded 12,542 participants without BMI data and 987 participants with BMI values of zero or greater than 60 kg/m^2^. Subsequently, participants were stratified into three categories based on their BMI: underweight (< 18.5 kg/m^2^), normal (18.5–24.9 kg/m^2^), and overweight or obese (≥ 25 kg/m^2^). For the CCI-based subgroup analysis, individuals were classified into the following two groups: those with no comorbidities (CCI = 0) and those with one or more comorbidities (CCI ≥ 1).

### Sensitivity analyses

We performed two sensitivity analyses to validate the robustness of our findings. First, after recognizing the potential variability in the level of medical care across different facilities, we employed a multivariable logistic regression model and adjusted for clustering within hospitals using a generalized estimating equation (Hubbard et al. 2010).

This approach was selected to account for the non-independence of observations within the same hospital, which is critical for obtaining unbiased estimates of treatment effects across diverse clinical settings. Second, to evaluate the influence of environmental conditions at the time of admission, we incorporated the “short-term local WBGT” into our main model as a covariate instead of the month of admission. The other covariates were the same as those used in the main analysis.

All statistical analyses were performed using Stata v18 (Stata Corp LLC, College Station, TX, USA). All tests were two sided, and significance was defined as a *P* value of < 0.05 or assessed using the 95% CIs.

## Results

**Fig. 1:**
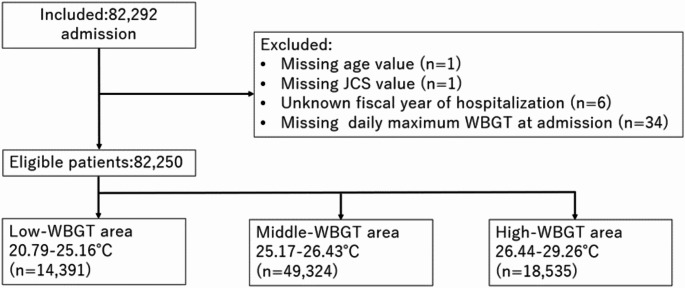
Patient flow diagram Abbreviations; WBGT, wet bulb globe temperature; JCS, Japan Coma Scale ・ Long-term local WBGT: The long-term average daily maximum WBGT for each prefecture during the study period ・ Local WBGT areas: We divided the 47 prefectures into three areas using the first and third quartiles of long-term local WBGT (low-WBGT area, 20.79–25.15 °C; middle-WBGT area, 25.16–26.43 °C; and high-WBGT area, 26.44–29.26 °C)

After applying the inclusion and exclusion criteria, we obtained data on 82,250 patients (from 1,702 hospitals among all 47 prefectures in Japan) admitted for the treatment of heat-related diseases (Fig. 1). The mean age was 63.2 (standard deviation, 25.0) years, and 63.7% were male. The median length of hospital stay was four days (interquartile range [IQR], 2–10), and the median hospitalization cost was $1,361 (IQR $818–$2,887). In the high local WBGT areas, the proportion of patients with acute kidney injury and acute liver failure tended to be higher yet with lower in-hospital mortality than that observed with the other WBGT areas (Table 1). The basic characteristics of patients stratified by age, sex, BMI, and CCI are presented in Supplemental Tables S1-S9. The males compared to females were relatively younger and exhibited a higher incidence of acute kidney injury. Patients in the overweight and obesity categories were younger than those in the underweight and normal weight categories.

**Table 1:** Baseline patient characteristics

	Total	Low-WBGT 20.79–25.16 ℃	Middle-WBGT 25.17–26.43 ℃	High-WBGT 26.44–29.26 ℃	*p*-value
	*N* = 82,250	*N* = 14,391	*N* = 49,324	*N* = 18,535	
**Personal-level variables**					
Age, average (standard deviation)	63.2(25.0)	64.6(25.5)	63.3(25.0)	61.9(24.8)	< 0.001
Age category					< 0.001
< 7	958(1.2)	164(1.1)	662(1.3)	132(0.7)	
7–17	6,656(8.1)	1,223(8.5)	3,795(7.7)	1,638(8.8)	
18–64	25,006(30.4)	3,908(27.2)	14,890(30.2)	6,208(33.5)	
64–79	21,226(25.8)	3,496(24.3)	13,053(26.5)	4,677(25.2)	
80 ≤	28,404(34.5)	5,600(38.9)	16,924(34.3)	5,880(31.7)	
Male	52,363(63.7)	8,779(61.0)	31,433(63.7)	12,151(65.6)	< 0.001
BMI category(kg/m^2^)					< 0.001
< 18.5	12,986(15.8)	2,193(15.2)	8,066(16.4)	2,727(14.7)	
18.5–24.9	40,999(49.8)	7,173(49.8)	24,236(49.1)	9,590(51.7)	
25.0–29.9	11,789(14.3)	2,035(14.1)	6,908(14.0)	2,846(15.4)	
30.0 ≤	2,947(3.6)	482(3.3)	1,755(3.6)	710(3.8)	
Missing	13,529(16.4)	2,508(17.4)	8,359(16.9)	2,662(14.4)	
Smoking	20,953(25.5)	3,379(23.5)	12,499(25.3)	5,075(27.4)	< 0.001
Missing	10,506(12.8)	1,857(12.9)	6,619(13.4)	2,030(11.0)	
Charlson Comorbidity Index	0(0–1)	0(0–1)	0(0–1)	0(0–1)	< 0.001
Myocardial infarction	809(1.0)	136(0.9)	499(1.0)	174(0.9)	0.61
Congestive heart failure	3,915(4.8)	776(5.4)	2,297(4.7)	842(4.5)	< 0.001
Peripheral vascular disease	670(0.8)	128(0.9)	403(0.8)	139(0.7)	0.38
Cerebrovascular disease	5,609(6.8)	1,049(7.3)	3,280(6.6)	1,280(6.9)	0.024
Dementia	5,321(6.5)	1,073(7.5)	3,196(6.5)	1,052(5.7)	< 0.001
Chronic Pulmonary disease	2,443(3.0)	425(3.0)	1,499(3.0)	519(2.8)	0.26
Rheumatic disease	627(0.8)	116(0.8)	348(0.7)	163(0.9)	0.054
Peptic ulcer disease	1,630(2.0)	350(2.4)	1,000(2.0)	280(1.5)	< 0.001
Mild liver disease	3,056(3.7)	462(3.2)	1,774(3.6)	820(4.4)	< 0.001
Diabetes without chronic complication	7,781(9.5)	1,349(9.4)	4,662(9.5)	1,770(9.5)	0.86
Diabetes with chronic complication	1,803(2.2)	300(2.1)	1,111(2.3)	392(2.1)	0.35
Hemiplegia/paraplegia	163(0.2)	32(0.2)	97(0.2)	34(0.2)	0.73
Renal disease	1,948(2.4)	340(2.4)	1,159(2.3)	449(2.4)	0.86
Malignancy	2,895(3.5)	604(4.2)	1,724(3.5)	567(3.1)	< 0.001
Moderate or severe liver disease	176(0.2)	24(0.2)	106(0.2)	46(0.2)	0.28
Metastatic solid tumor	457(0.6)	111(0.8)	265(0.5)	81(0.4)	< 0.001
AIDS/HIV	9(0.0)	0(0.0)	9(0.0)	0(0.0)	0.050
Mental disorder	5,341(6.5)	944(6.6)	3,219(6.5)	1,178(6.4)	0.68
**Admission year and month**					
Fiscal year					< 0.001
2011	6,784(8.2)	1,130(7.9)	4,196(8.5)	1,458(7.9)	
2012	6,507(7.9)	1,266(8.8)	3,810(7.7)	1,431(7.7)	
2013	8,823(10.7)	1,114(7.7)	5,479(11.1)	2,230(12.0)	
2014	6,453(7.8)	1,197(8.3)	3,901(7.9)	1,355(7.3)	
2015	9,316(11.3)	1,676(11.6)	5,577(11.3)	2,063(11.1)	
2016	9,259(11.3)	1,500(10.4)	5,138(10.4)	2,621(14.1)	
2017	8,607(10.5)	1,518(10.5)	4,885(9.9)	2,204(11.9)	
2018	15,717(19.1)	2,727(18.9)	9,876(20.0)	3,114(16.8)	
2019	10,784(13.1)	2,263(15.7)	6,462(13.1)	2,059(11.1)	
Month					< 0.001
May	2,771(3.4)	597(4.1)	1,488(3.0)	686(3.7)	
June	5,236(6.4)	950(6.6)	3,136(6.4)	1,150(6.2)	
July	34,833(42.4)	5,789(40.2)	21,224(43.0)	7,820(42.2)	
August	35,032(42.6)	6,368(44.2)	20,925(42.4)	7,739(41.8)	
September	4,378(5.3)	687(4.8)	2,551(5.2)	1,140(6.2)	
**Severity**					
Japan Coma Scale category at admission					< 0.001
Alert	56,390(68.6)	9,542(66.3)	33,670(68.3)	13,178(71.1)	
Dizzy	17,408(21.2)	3,330(23.1)	10,438(21.2)	3,640(19.6)	
Drowsy	4,474(5.4)	792(5.5)	2,729(5.5)	953(5.1)	
Come	3,978(4.8)	727(5.1)	2,487(5.0)	764(4.1)	
Acute liver failure	1,834(2.2)	282(2.0)	1,090(2.2)	462(2.5)	0.005
Acute kidney injury	7,936(9.6)	1,151(8.0)	4,663(9.5)	2,122(11.4)	< 0.001
Disseminated intravascular coagulation	1,707(2.1)	313(2.2)	1,022(2.1)	372(2.0)	0.57
**Intervention**					
Intensive care unit	3,467(4.2)	353(2.5)	2,463(5.0)	651(3.5)	< 0.001
High care unit	7,778(9.5)	1,455(10.1)	4,913(10.0)	1,410(7.6)	< 0.001
Ambulance use	54,432(66.2)	9,283(64.5)	33,334(67.6)	11,815(63.7)	< 0.001
Catecholamine use	2,516(3.1)	469(3.3)	1,542(3.1)	505(2.7)	0.008
Mechanical ventilation	2,257(2.7)	358(2.5)	1,482(3.0)	417(2.2)	< 0.001
Renal replacement therapy	876(1.1)	133(0.9)	526(1.1)	217(1.2)	0.097
**Outcomes**					
In-hospital mortality	2,044(2.5)	456(3.2)	1,194(2.4)	394(2.1)	< 0.001
Length of hospital stay, median (IQR)	4(2–10)	4(2–10)	4(2–10)	4(2–9)	< 0.001
Hospitalization cost, median (IQR)	1361(818–2887)	1384(827–2937)	1403.087(841–2967)	1234.06(762–2631)	< 0.001

Abbreviations; WBGT, wet bulb globe temperature; BMI, body mass index; IQR, interquartile range; AIDS/HIV, acquired immunodeficiency syndrome/human immunodeficiency virus. Data are presented as numbers (%) unless otherwise indicated

・ Long-term local WBGT: The long-term average daily maximum WBGT for each prefecture during the study period

・ Local WBGT areas: We divided the 47 prefectures into three areas using the first and third quartiles of long-term local WBGT (low-WBGT area, 20.79–25.15 °C; middle-WBGT area, 25.16–26.43 °C; and high-WBGT area, 26.44–29.26 °C)

**Fig. 2:**
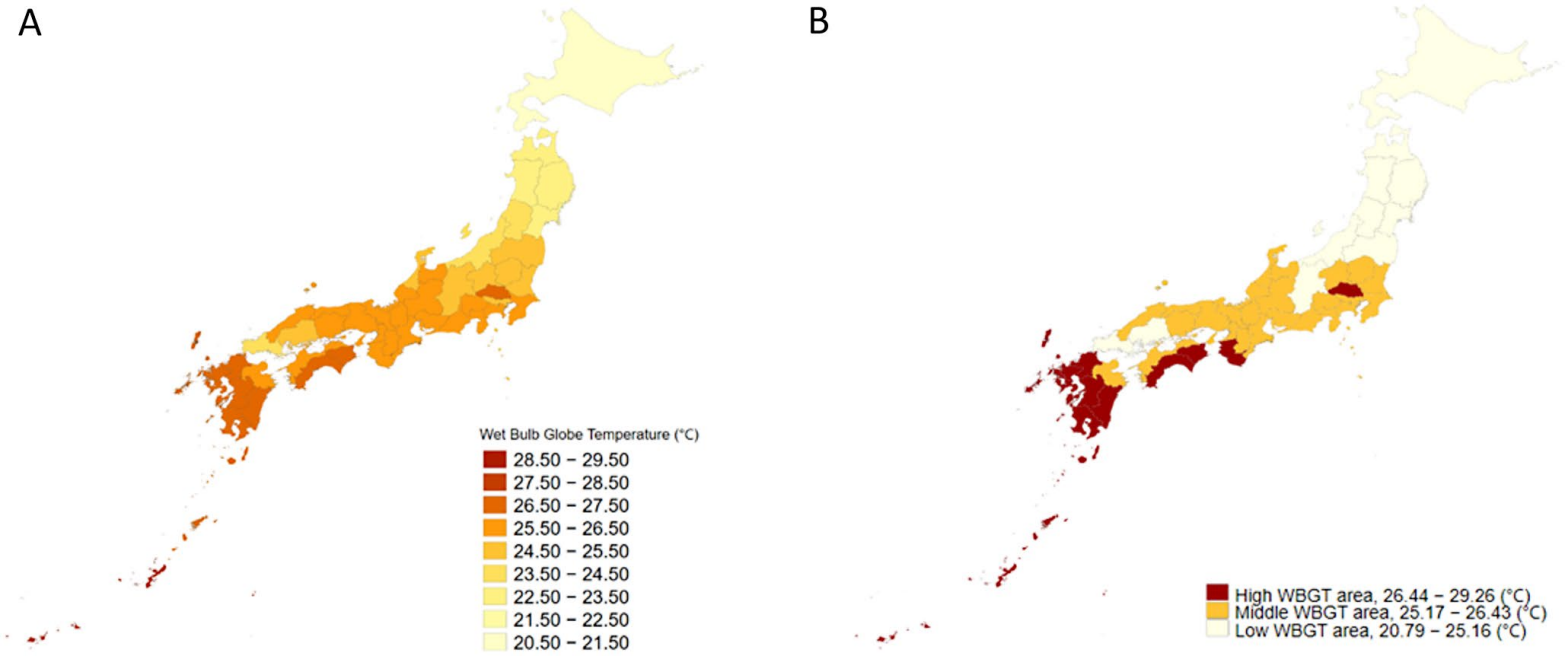
**A** Distribution of long-term local WBGT in 47 prefectures. **B** Distribution of local WBGT areas. ・ Long-term local WBGT: The long-term average daily maximum WBGT for each prefecture during the study period. ・ Local WBGT areas: We divided the 47 prefectures into three areas using the first and third quartiles of long term local WBGT (low-WBGT area, 20.79–25.15 °C; middle-WBGT area, 25.16–26.43 °C; and high-WBGT area, 26.44–29.26 °C)

Figure 2A illustrates the geographical distribution of the long-term local WBGTs, representing the average daily maximum WBGT during the study period across all the 47 prefectures of Japan. The median long-term local WBGT was 26.0 (IQR: 25.2–26.4) ℃. Details for each prefecture are summarized in Supplemental Table S10. Figure 2B displays the categorization of regions into the three local WBGT areas based on the first and third quartiles of the long-term local WBGT. Figure 3A presents the distribution of the proportion of admissions at each short-term local WBGT (daily maximum WBGT on the day of admission) relative to the total admissions within the same WBGT category over the observation period. Figure 3B presents the distribution of the proportion of deaths at each short-term local WBGT relative to the total deaths within the same WBGT category over the observation period. The distributions shifted according to the local climate. For example, in the low-WBGT areas, more admissions and deaths due to heat-related diseases occurred in the range of lower WBGT.

**Fig. 3A:**
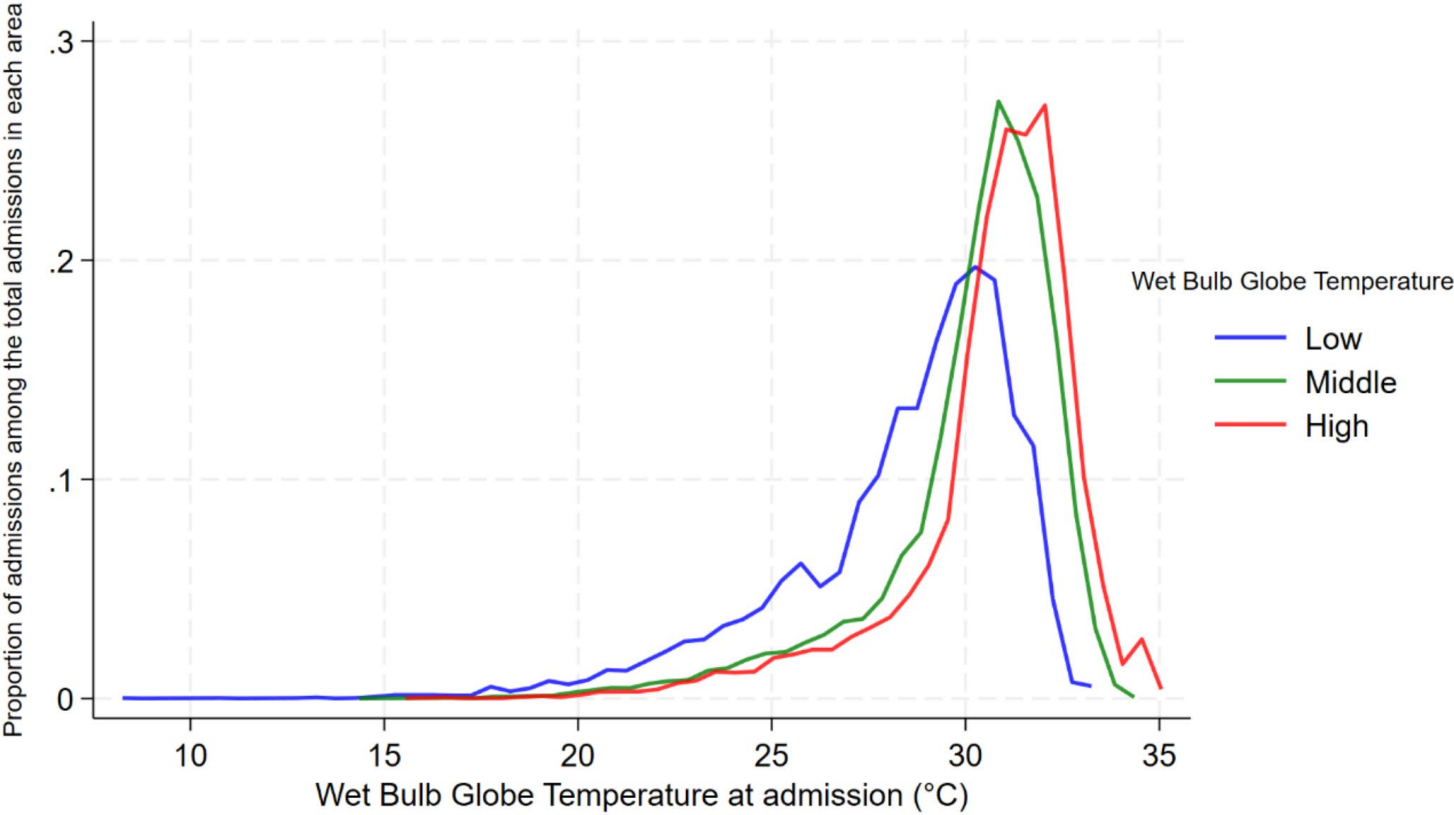
Proportion of admissions at each short-term local WBGT (daily maximum WBGT on the day of admission) relative to the total admissions within the same WBGT category over the observation period Figure 3A illustrates the proportion of admissions at each short-term local WBGT (representing the daily maximum WBGT for each prefecture on the day of hospital admission), calculated as the number of admissions within each WBGT category divided by the total number of admissions in the same WBGT category over the observation period

**Fig. 3B:**
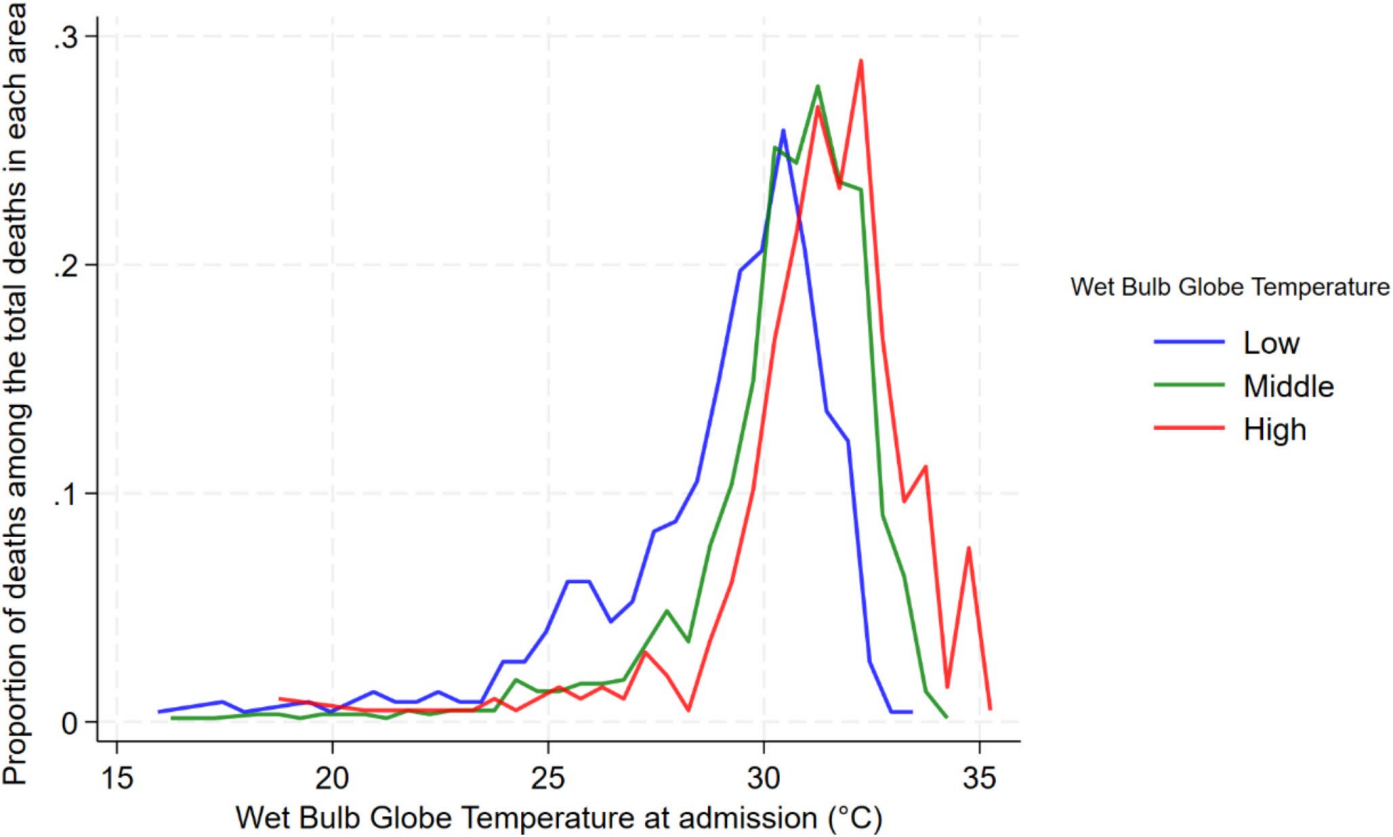
Proportion of deaths at each short-term local WBGT (daily maximum WBGT on the day of admission) relative to the total deaths within the same WBGT category over the observation period Abbreviation; WBGT, wet bulb globe temperature Figure 3B illustrates the proportion of deaths at each short-term local WBGT (representing the daily maximum WBGT for each prefecture on the day of hospital admission), calculated as the number of deaths within each WBGT category divided by the total number of deaths in the same WBGT category over the observation period Local WBGT areas: We divided the 47 prefectures into three areas using the first and third quartiles of long-term local WBGT (low-WBGT area, 20.79–25.15 °C; middle-WBGT area, 25.16–26.43 °C; and high-WBGT area, 26.44–29.26 °C)

Table 2 presents the study outcomes. In-hospital mortality was 2.1%, 2.4%, and 3.2% in the high-WBGT, middle-WBGT, and low-WBGT areas, respectively. In the multivariable logistic regression analysis, the low-WBGT area had a higher in-hospital mortality than that had by the high-WBGT area, with an OR of 1.32 (95% CI, 1.15–1.52). However, we found no significant difference in in-hospital mortality between the middle-WBGT and high-WBGT areas (OR of 1.00 [95% CI, 0.89–1.12]).

**Table 2:** Multivariable logistic regression analysis results for in-hospital mortality

	In-hospital mortality (%)	Adjusted odds ratio (95% CI)	*p* value
Low-WBGT area (20.79–25.16 ℃)	456/14,391 (3.2)	1.32 (1.15–1.52)	< 0.001
Middle-WBGT area (25.17–26.43 ℃)	1,192/49,324 (2.4)	1.00 (0.89–1.12)	0.948
High-WBGT area (26.44–29.26 ℃)	394/18,535 (2.1)	Ref	

Abbreviations; WBGT, wet bulb globe temperature; CI, confidence interval

・ Long-term local WBGT: The long-term average daily maximum WBGT for each prefecture during the study period

・ Local WBGT areas: We divided the 47 prefectures into three areas using the first and third quartiles of long-term local WBGT (low-WBGT area, 20.79–25.15 °C; middle-WBGT area, 25.16–26.43 °C; and high-WBGT area, 26.44–29.26 °C)

Supplemental Table S11 shows the ORs of the covariates in the multivariable regression analysis for in-hospital mortality other than the WBGT categories. Age, CCI, individuals with underweight (< 18.5 kg/m^2^) and obesity (> 30.0 kg/m^2^), smoking, transportation by ambulance, and hospitalization in July and August were independently associated with in-hospital mortality.

Table 3 displays the results of the subgroup analyses. A higher in-hospital mortality in low-WBGT areas, similar to the main analysis, was observed among older adults (aged 75 years and above) and groups classified as underweight and normal weight. However, no significant differences in in-hospital mortality were found among younger individuals (under 75 years) and those in the obese and overweight categories in low-WBGT areas. The findings of the remaining subgroups were consistent in direction and magnitude with those of the main analysis, irrespective of the sex or CCI score.

**Table 3:** Subgroup analyses results

	In-hospital mortality (%)		Adjusted odds ratio (95% CI)	*p* value
**Aged ≥ 75 years**				
Low-WBGT area	338/7,174	4.7	1.35 (1.14–1.60)	< 0.001
Middle-WBGT area	819/22,586	3.6	1.00 (0.87–1.16)	0.961
High-WBGT area	264/7,835	3.4	Ref	
**Aged < 75 years**				
Low-WBGT area	118/7,217	1.6	1.27 (0.99–1.65)	0.065
Middle-WBGT area	375/26,738	1.4	0.97(0.79–1.20)	0.801
High-WBGT area	130/10,700	1.2	Ref	
**Male**				
Low-WBGT area	253/8,779	2.9	1.29 (1.07–1.55)	0.007
Middle-WBGT area	698/ 31,433	2.2	0.98 (0.84–1.14)	0.795
High-WBGT area	236/12,151	1.9	Ref	
**Female**				
Low-WBGT area	203/5,612	3.6	1.39 (1.12–1.72)	0.003
Middle-WBGT area	496/17,891	2.8	1.02 (0.85–1.23)	0.802
High-WBGT area	158/6,384	2.5	Ref	
**Underweight (BMI < 18.5 kg/m**^**2**^)				
Low-WBGT area	100/2,193	4.6	1.66 (1.22–2.27)	0.001
Middle-WBGT area	267/8,066	3.3	1.18 (0.91–1.54)	0.216
High-WBGT area	74/2,727	2.7	Ref	
**Normal (18.5 kg/m**^**2**^ **≤ BMI < 25 kg/m**^**2**^)				
Low-WBGT area	165/7,168	2.3	1.42 (1.13–1.80)	0.003
Middle-WBGT area	396/24,210	1.6	1.07 (0.88–1.31)	0.501
High-WBGT area	134/9,578	1.4	Ref	
**Overweight and obese (25 kg/m**^**2**^ **≤ BMI)**				
Low-WBGT area	46/2,522	1.8	1.10 (0.73–1.64)	0.660
Middle-WBGT area	129/8,689	1.5	0.93 (0.67–1.29)	0.653
High-WBGT area	52/3,568	1.5	Ref	
**No comorbidities (CCI = 0)**				
Low-WBGT area	242/9,093	2.7	1.25 (1.03–1.51)	0.024
Middle-WBGT area	673/32,026	2.1	0.98 (0.83–1.14)	0.763
High-WBGT area	218/12,155	1.8	Ref	
**One or more comorbidities (CCI ≥ 1)**				
Low-WBGT area	214/5,298	4.0	1.37 (1.12–1.69)	0.003
Middle-WBGT area	521/17,298	3.0	1.01 (0.84–1.20)	0.942
High-WBGT area	176/6,380	2.8	Ref	

Abbreviations; WBGT, wet bulb globe temperature; BMI, body mass index; CCI, Charlson Comorbidity Index; CI, confidence interval

・ Long-term local WBGT: The long-term average daily maximum WBGT for each prefecture during the study period

・ Local WBGT areas: We divided the 47 prefectures into three areas using the first and third quartiles of long-term local WBGT (low-WBGT area, 20.79–25.15 °C; middle-WBGT area, 25.16–26.43 °C; and high-WBGT area, 26.44–29.26 °C)

Table 4 presents the results of the sensitivity analyses. The two sensitivity analyses results were consistent with those of the main analysis.

**Table 4:** Sensitivity analyses results

	In-hospital mortality (%)		Adjusted odds ratio (95% CI)	*p* value
**Analysis incorporating short-term WBGT as a covariate instead of the monthly variable**				
Low-WBGT area	456/14,391	3.2	1.50 (1.30–1.74)	< 0.001
Middle-WBGT area	1,192/49,324	2.4	1.04 (0.92–1.17)	0.555
High-WBGT area	394/18,535	2.1	Ref	
**Multivariable logistic regression model fitted with a generalized estimating equation**				
Low-WBGT area	456/14,391	3.2	1.32 (1.10–1.59)	< 0.001
Middle-WBGT area	1,192/49,324	2.4	0.99 (0.85–1.17)	0.941
High-WBGT area	394/18,535	2.1	Ref	

Abbreviations; WBGT, wet bulb globe temperature; CI, confidence interval

・ Long-term local WBGT: The long-term average daily maximum WBGT for each prefecture during the study period

・ Local WBGT areas: We divided the 47 prefectures into three areas using the first and third quartiles of long-term local WBGT (low-WBGT area, 20.79–25.15 °C; middle-WBGT area, 25.16–26.43 °C; and high-WBGT area, 26.44–29.26 °C)

・ Short-term local WBGT: The daily maximum WBGT of each prefecture at the day of admission

## Discussion and conclusions

In this nationwide observational study using climate and clinical individual data, we found that in-hospital death was more likely to occur in patients with heat-related diseases in low WBGT areas compared with those in high WBGT areas. Our findings underscore the critical need for increased awareness and preventive measures against heat-related diseases, especially in areas with relatively low WBGT.

Our findings are supported by previous studies that reported that a relatively cool regional climate was associated with increased heat-related disease mortality and emergency room visits. In the United States, the adverse effects of heat waves on mortality were more pronounced in the northern regions than in the southern regions (Curriero et al. 2002; Anderson and Bell 2011). Moreover, emergency room visits due to hyperthermia occurred in higher latitudes than in lower latitudes in the United States (Saha et al. 2015). Similarly, in Europe, differences in heat stress tolerance by latitude have been observed among the major cities, such as London, Paris, Rome, Budapest, Barcelona, and Krakow, as well as within Poland (Blazejczyk and McGregor 2008; Ward et al. 2016; Kuchcik 2021). In Japan, several studies have also reported that local temperature and age are associated with the number of emergency transports for heat-related illness (Miyatake et al. 2012; Ueno et al. 2021). The evidence on ambient temperature was also true for WBGT; if the populations were exposed to the same heat conditions, an inverse relationship was observed between the average daily maximum WBGT in the area and the number of heat-related emergency transportations (Ueno et al. 2021; Oka et al. 2022). Although these studies have significantly contributed to the understanding of the increased vulnerability of residents in cooler areas during the summer, they primarily focused on aggregate data without delving into individual-level factors, such as age, obesity, and comorbidities, as key determinants of heat vulnerability. Our study advances this field by demonstrating that residents in cooler areas compared with those in warmer areas face higher risks of in-hospital mortality, even when individual-level risk factors are considered.

In the present study, age, CCI, and underweight or obesity were significantly associated with in-hospital mortality among patients with heat-related diseases. Many previous studies have indicated that age, obesity, and comorbidities were associated with vulnerability to heat stress (Bouchama and Knochel 2002; Shimazaki et al. 2020; Westwood et al. 2021). Although studies on the association between underweight and heat-related illnesses are scarce (Xu et al. 2019), mortality rates are generally high among patients who are underweight (Visscher et al. 2000; Roh et al. 2014). Previous studies have shown that patients with physical illnesses, dementia, and mental illnesses were at an increased risk of developing heat-related diseases (Schmeltz and Gamble 2017). In the present study, mental illness was included as a covariate, but it was not statistically significantly associated with in-hospital mortality.

This vulnerability of residents in cooler areas can be generally explained by the following two reasons. First, people living in warmer areas compared with those in cooler areas are more familiar with adopting strategies to cope with hot climates (for example, using technologies such as air conditioning) (Sera et al. 2020). Second, individuals in warmer areas have a higher heat tolerance due to their acclimatization to high temperatures (Lee et al. 2014; Oka and Hijioka 2021).

In the present study, the main subgroup analyses based on age, sex, BMI, and CCI revealed that in-hospital mortality was significantly higher in the low WBGT areas than in the other areas, but the difference was not significant in the subgroup of those younger than 75 years or in those classified as overweight and obese compared with those aged 75 years and above and those who are underweight, respectively. In the context of individuals aged 75 years and younger, this absence of increased heat vulnerability in low WBGT areas may reflect an age-related interaction. Moreover, unlike the underweight or normal weight groups, the overweight and obese group predominantly comprises individuals aged 18–64 years. Consequently, the absence of a significant mortality difference between the overweight and obese group and the other weight groups is more likely attributable to age differences than to BMI. A previous population-based systematic review suggested that males compared to females have higher lifetime heat-related disease morbidity and mortality (Gifford et al. 2019), yet the sex modification effect was not evident in hospitalized patients in the present study.

A comprehensive public health intervention tailored to the local climate is essential to mitigate the differences in heat-related disease mortality influenced by the climate of the residential area. The WHO European Office has already proposed Heat Health Action Plans as a comprehensive framework, which includes accurate and timely alert systems (Heat Health Warning System), appropriate information dissemination, reduction of indoor heat exposure, and particular care for the vulnerable population (Matthies et al. 2008; Martinez et al. 2019, 2022). In Japan, since the enactment of the Climate Change Adaptation Act in 2018, the government has been implementing soft and hard measures at both the national and local levels to mitigate the risks associated with extreme heat (Ministry of the Environment, Japan 2020). Soft measures include heatstroke alerts and public awareness campaigns while hard measures involve air conditioner subscription services and “urban greening” promotion (Kim et al. 2023). However, the evidence quantifying the effectiveness of these measures remains insufficient (Rao-Skirbekk et al. 2023). Further research is needed to assess the preventive effects and economic impacts of these measures. Additionally, it is essential for policymakers, researchers, healthcare professionals, and other stakeholders to collaborate to develop and implement evidence-based interventions.

This study had some limitations. First, we categorized patients into three groups based on the WBGT of their admission sites; however, we did not consider individual exposure to WBGT. Second, we used the nationwide in-hospital database, and this database does not cover all hospitals in Japan. However, the high coverage of tertiary hospitals (approximately 90%) would cover most patients with heat-related diseases, especially with a severe disease. Third, potential unmeasured confounders, including cooling methods, time from disease onset to cooling, medications, socioeconomic status, and occupational vulnerability, might have affected the in-hospital mortality.

In summary, in-hospital mortality in patients with heat-related diseases may be influenced by the average daily maximum WBGT at the prefectural level. In addressing heat-related diseases, policymakers should consider the different susceptibility to WBGT at a local level. Our findings suggest that more support should be provided to communities in cooler areas to help prevent deaths due to heat-related diseases.

## Electronic supplementary material

Below is the link to the electronic supplementary material.

Supplementary Material 1

## Data Availability

The datasets generated and analyzed for this study are available upon reasonable request.
