# Supplementary material for: In-hospital mortality of heat-related disease associated with wet bulb globe temperature: a Japanese nationwide inpatient data analysis
Source: Int J Biometeorol. 2025 Mar 4;69(4):873–84. doi: 10.1007/s00484-025-02867-x (PMC11947014; doi:10.1007/s00484-025-02867-x)
Supplement: Supplementary file 1 — Supplementary Material 1 [file 484_2025_2867_MOESM1_ESM.docx]

**Supplemental Materials**

**List of Contents**

**Table S1** Baseline characteristics of patients aged 75 years and older

**Table S2** Baseline characteristics of patients aged younger than 75 years

**Table S3** Baseline characteristics of male patients

**Table S4** Baseline characteristics of female patients

**Table S5** Baseline characteristics of underweight patients

**Table S6** Baseline characteristics of patients with normal weight

**Table S7** Baseline characteristics of patients with overweight or obesity

**Table S8** Baseline characteristics of patients with no comorbidities (CCI = 0)

**Table S9** Baseline characteristics of patients with one or more comorbidities (CCI ≥ 1)

**Table S10** Long-term average Wet-Bulb Globe Temperature (°C) across 47 prefectures.

**Table S11** Relative risks of covariates other than WBGT categories in the multivariable regression analysis predicting mortality

**Table S1** Baseline characteristics of patients aged 75 years and older

|  | **Total** | **Low-WBGT**  **20.9-25.1 ℃** | **Middle-WBGT**  **25.2-26.4 ℃** | **High-WBGT**  **26.5-29.2 ℃** | **p-value** |
| --- | --- | --- | --- | --- | --- |
|  | N=37,595 | N=7,174 | N=22,586 | N=7,835 |  |
| **Personal-level variables** | |  |  |  |  |
| Age, average (standard deviation) | 84.0(5.5) | 84. (5.6) | 83.8(5.5) | 83. (5.4) | <0.001 |
| Age category |  |  |  |  |  |
| 75 - 79 | 9,191(24.4) | 1,574(21.9) | 5,662(25.1) | 1,955(25.0) | <0.001 |
| 80 ≤ | 28,404(75.6) | 5,600(78.1) | 16,924(74.9) | 5,880(75.0) |  |
| Male | 18,187(48.4) | 3,355(46.8) | 10,982(48.6) | 3,850(49.1) | 0.007 |
| BMI category(kg/m^2^) |  |  |  |  |  |
| <18.5 | 7,402(19.7) | 1,343(18.7) | 4,520(20.0) | 1,539(19.6) | <0.001 |
| 18.5 – 24.9 | 18,949(50.4) | 3,644(50.8) | 11,250(49.8) | 4,055(51.8) |  |
| 25.0 – 29.9 | 4,275(11.4) | 839(11.7) | 2,489(11.0) | 947(12.1) |  |
| 30.0 ≤ | 603(1.6) | 126(1.8) | 337(1.5) | 140(1.8) |  |
| Missing | 6,366(16.9) | 1,222(17.0) | 3,990(17.7) | 1,154(14.7) |  |
| Smoking | 6,463(17.2) | 1,155(16.1) | 3,982(17.6) | 1,326(16.9) | <0.001 |
| Missing | 4,801(12.8) | 899(12.5) | 3,034(13.4) | 868(11.1) |  |
| Charlson Comorbidity Index | 1(0-1) | 1(0-1) | 0(0-1) | 1(0-1) | <0.001 |
| Myocardial infarction | 579(1.5) | 104(1.4) | 351(1.6) | 124(1.6) | 0.78 |
| Congestive  heart failure | 3,156(8.4) | 644(9.0) | 1,854(8.2) | 658(8.4) | 0.12 |
| Peripheral  vascular disease | 507(1.3) | 103(1.4) | 301(1.3) | 103(1.3) | 0.77 |
| Cerebrovascular disease | 4,047(10.8) | 815(11.4) | 2,332(10.3) | 900(11.5) | 0.003 |
| Dementia | 4,750(12.6) | 976(13.6) | 2,836(12.6) | 938(12.0) | 0.009 |
| Chronic  Pulmonary  disease | 1,666(4.4) | 303(4.2) | 1,014(4.5) | 349(4.5) | 0.63 |
| Rheumatic disease | 413(1.1) | 72(1.0) | 236(1.0) | 105(1.3) | 0.067 |
| Peptic ulcer disease | 979(2.6) | 241(3.4) | 580(2.6) | 158(2.0) | <0.001 |
| Mild liver disease | 1,101(2.9) | 184(2.6) | 649(2.9) | 268(3.4) | 0.006 |
| Diabetes without chronic complication | 4,446(11.8) | 860(12.0) | 2,652(11.7) | 934(11.9) | 0.82 |
| Diabetes with chronic complication | 912(2.4) | 182(2.5) | 559(2.5) | 171(2.2) | 0.28 |
| Hemiplegia/paraplegia | 72(0.2) | 16(0.2) | 39(0.2) | 17(0.2) | 0.59 |
| Renal disease | 1,274(3.4) | 237(3.3) | 734(3.2) | 303(3.9) | 0.031 |
| Malignancy | 2,105(5.6) | 458(6.4) | 1,234(5.5) | 413(5.3) | 0.005 |
| Moderate or severe liver disease | 67(0.2) | 10(0.1) | 43(0.2) | 14(0.2) | 0.67 |
| Metastatic solid tumor | 289(0.8) | 73(1.0) | 163(0.7) | 53(0.7) | 0.025 |
| AIDS/HIV | 1(0.0) | 0(0.0) | 1(0.0) | 0(0.0) | 0.72 |
| Mental disorder | 1,925(5.1) | 392(5.5) | 1,163(5.1) | 370(4.7) | 0.11 |
| **Admission year and month** | |  |  |  |  |
| Fiscal year |  |  |  |  | <0.001 |
| 2011 | 2,755(7.3) | 515(7.2) | 1,723(7.6) | 517(6.6) |  |
| 2012 | 2,579(6.9) | 545(7.6) | 1,547(6.8) | 487(6.2) |  |
| 2013 | 3,800(10.1) | 542(7.6) | 2,387(10.6) | 871(11.1) |  |
| 2014 | 2,612(6.9) | 545(7.6) | 1,556(6.9) | 511(6.5) |  |
| 2015 | 4,454(11.8) | 871(12.1) | 2,634(11.7) | 949(12.1) |  |
| 2016 | 4,259(11.3) | 719(10.0) | 2,403(10.6) | 1,137(14.5) |  |
| 2017 | 3,936(10.5) | 739(10.3) | 2,228(9.9) | 969(12.4) |  |
| 2018 | 7,665(20.4) | 1,421(19.8) | 4,825(21.4) | 1,419(18.1) |  |
| 2019 | 5,535(14.7) | 1,277(17.8) | 3,283(14.5) | 975(12.4) |  |
| Month |  |  |  |  | <0.001 |
| May | 1,113(3.0) | 281(3.9) | 585(2.6) | 247(3.2) | <0.001 |
| June | 2,239(6.0) | 434(6.0) | 1,367(6.1) | 438(5.6) |  |
| July | 16,544(44.0) | 2,934(40.9) | 10,117(44.8) | 3,493(44.6) |  |
| August | 16,072(42.8) | 3,236(45.1) | 9,547(42.3) | 3,289(42.0) |  |
| September | 1,627(4.3) | 289(4.0) | 970(4.3) | 368(4.7) |  |
| **Severity** |  |  |  |  |  |
| Japan Coma Scale category at admission |  |  |  |  | <0.001 |
| Alert | 22,902(60.9) | 4,205(58.6) | 13,714(60.7) | 4,983(63.6) |  |
| Dizzy | 10,403(27.7) | 2,095(29.2) | 6,221(27.5) | 2,087(26.6) |  |
| Drowsy | 2,427(6.5) | 469(6.5) | 1,501(6.6) | 457(5.8) |  |
| Come | 1,863(5.0) | 405(5.6) | 1,150(5.1) | 308(3.9) |  |
| Acute liver failure | 553(1.5) | 84(1.2) | 346(1.5) | 123(1.6) | 0.062 |
| Acute kidney injury | 1,855(4.9) | 302(4.2) | 1,112(4.9) | 441(5.6) | <0.001 |
| Disseminated intravascular coagulation | 753(2.0) | 156(2.2) | 446(2.0) | 151(1.9) | 0.50 |
| **Intervention** |  |  |  |  |  |
| Intensive care unit | 1,533(4.1) | 165(2.3) | 1,120(5.0) | 248(3.2) | <0.001 |
| High care unit | 3,729(9.9) | 773(10.8) | 2,321(10.3) | 635(8.1) | <0.001 |
| Ambulance use | 26,464(70.4) | 4,837(67.4) | 16,171(71.6) | 5,456(69.6) | <0.001 |
| Catecholamine use | 1,392(3.7) | 278(3.9) | 835(3.7) | 279(3.6) | 0.59 |
| Mechanical  ventilation | 1,009(2.7) | 184(2.6) | 651(2.9) | 174(2.2) | 0.006 |
| Renal replacement therapy | 342(0.9) | 56(0.8) | 207(0.9) | 79(1.0) | 0.34 |
| **Outcomes** |  |  |  |  |  |
| In-hospital mortality | 1,421(3.8) | 338(4.7) | 819(3.6) | 264(3.4) | <0.001 |
| Length of hospital stay, median (IQR) | 7(3-16) | 7(3-17) | 7(3-16) | 7(3-16) | 0.81 |
| Hospitalization cost, median (IQR) | 2025  (1118-4213) | 1998  (1111-4249) | 2060  (1144-4232) | 1962  (1043-4132) | <0.001 |

Abbreviations; WBGT, wet bulb globe temperature; BMI, body mass index; IQR, interquartile range; AIDS/HIV, acquired immunodeficiency syndrome/human immunodeficiency virus. Data are presented as numbers (%) unless otherwise indicated.

- Long-term local WBGT: The long-term average daily maximum WBGT for each prefecture during the study period.
- Local WBGT areas: We divided the 47 prefectures into three areas using the first and third quartiles of long-term local WBGT (low-WBGT area, 20.79-25.15 °C; middle-WBGT area, 25.16-26.43 °C; and high-WBGT area, 26.44-29.26 °C).

**Table S2** Baseline characteristics of patients aged younger than 75 years

|  | **Total** | **Low-WBGT**  **20.9-25.1 ℃** | | **Middle-WBGT**  **25.2-26.4 ℃** | **High-WBGT**  **26.5-29.2 ℃** | **p-value** |
| --- | --- | --- | --- | --- | --- | --- |
|  | N=44,655 | N=7,217 | | N=26,738 | N=10,700 |  |
| **Personal-level variables** | |  | |  |  |  |
| Age, average (standard deviation) | 45.7(21.4) | 44.8 (21.9) | | 45.9 (21.5) | 45.8 (20.9) | <0.001 |
| Age category |  |  | |  |  | <0.001 |
| 0-6 | 958(2.1) | 164(2.3) | | 662(2.5) | 132(1.2) |  |
| 7-17 | 6,656(14.9) | 1,223(16.9) | | 3,795(14.2) | 1,638(15.3) |  |
| 18-64 | 25,006(56.0) | 3,908(54.1) | | 14,890(55.7) | 6,208(58.0) |  |
| 65-75 | 12,035(27.0) | 1,922(26.6) | | 7,391(27.6) | 2,722(25.4) |  |
| Male | 34,176(76.5) | 5,424(75.2) | | 20,451(76.5) | 8,301(77.6) | <0.001 |
| BMI category(kg/m^2^) |  |  | |  |  |  |
| <18.5 | 5,584(12.5) | 850(11.8) | | 3,546(13.3) | 1,188(11.1) | <0.001 |
| 18.5 – 24.9 | 22,050(49.4) | 3,529(48.9) | | 12,986(48.6) | 5,535(51.7) |  |
| 25.0 – 29.9 | 7,514(16.8) | 1,196(16.6) | | 4,419(16.5) | 1,899(17.7) |  |
| 30.0 ≤ | 2,344(5.2) | 356(4.9) | | 1,418(5.3) | 570(5.3) |  |
| Missing | 7,163(16.0) | 1,286(17.8) | | 4,369(16.3) | 1,508(14.1) |  |
| Smoking | 14,490(32.4) | 2,224(30.8) | | 8,517(31.9) | 3,749(35.0) | <0.001 |
| Missing | 5,705(12.8) | 958(13.3) | | 3,585(13.4) | 1,162(10.9) |  |
| Charlson Comorbidity Index | 0(0-0) | 0(0-0) | | 0(0-0) | 0(0-0) | 0.24 |
| Myocardial infarction | 230(0.5) | 32(0.4) | | 148(0.6) | 50(0.5) | 0.37 |
| Congestive  heart failure | 759(1.7) | 132(1.8) | | 443(1.7) | 184(1.7) | 0.59 |
| Peripheral  vascular disease | 163(0.4) | 25(0.3) | | 102(0.4) | 36(0.3) | 0.78 |
| Cerebrovascular disease | 1,562(3.5) | 234(3.2) | | 948(3.5) | 380(3.6) | 0.43 |
| Dementia | 571(1.3) | 97(1.3) | | 360(1.3) | 114(1.1) | 0.079 |
| Chronic  Pulmonary  disease | 777(1.7) | 122(1.7) | | 485(1.8) | 170(1.6) | 0.30 |
| Rheumatic disease | 214(0.5) | 44(0.6) | | 112(0.4) | 58(0.5) | 0.064 |
| Peptic ulcer disease | 651(1.5) | | 109(1.5) | 420(1.6) | 122(1.1) | 0.007 |
| Mild liver disease | 1,955(4.4) | 278(3.9) | | 1,125(4.2) | 552(5.2) | <0.001 |
| Diabetes without chronic complication | 3,335(7.5) | 489(6.8) | | 2,010(7.5) | 836(7.8) | 0.031 |
| Diabetes with chronic complication | 891(2.0) | 118(1.6) | | 552(2.1) | 221(2.1) | 0.057 |
| Hemiplegia/paraplegia | 91(0.2) | 16(0.2) | | 58(0.2) | 17(0.2) | 0.50 |
| Renal disease | 674(1.5) | 103(1.4) | | 425(1.6) | 146(1.4) | 0.22 |
| Malignancy | 790(1.8) | 146(2.0) | | 490(1.8) | 154(1.4) | 0.007 |
| Moderate or severe liver disease | 109(0.2) | 14(0.2) | | 63(0.2) | 32(0.3) | 0.34 |
| Metastatic solid tumor | 168(0.4) | 38(0.5) | | 102(0.4) | 28(0.3) | 0.017 |
| AIDS/HIV | 8(0.0) | 0(0.0) | | 8(0.0) | 0(0.0) | 0.069 |
| Mental disorder | 3,416(7.6) | 552(7.6) | | 2,056(7.7) | 808(7.6) | 0.90 |
| **Admission year and month** | |  | |  |  |  |
| Fiscal year |  |  | |  |  | <0.001 |
| 2011 | 4,029(9.0) | 615(8.5) | | 2,473(9.2) | 941(8.8) |  |
| 2012 | 3,928(8.8) | 721(10.0) | | 2,263(8.5) | 944(8.8) |  |
| 2013 | 5,023(11.2) | 572(7.9) | | 3,092(11.6) | 1,359(12.7) |  |
| 2014 | 3,841(8.6) | 652(9.0) | | 2,345(8.8) | 844(7.9) |  |
| 2015 | 4,862(10.9) | 805(11.2) | | 2,943(11.0) | 1,114(10.4) |  |
| 2016 | 5,000(11.2) | 781(10.8) | | 2,735(10.2) | 1,484(13.9) |  |
| 2017 | 4,671(10.5) | 779(10.8) | | 2,657(9.9) | 1,235(11.5) |  |
| 2018 | 8,052(18.0) | 1,306(18.1) | | 5,051(18.9) | 1,695(15.8) |  |
| 2019 | 5,249(11.8) | 986(13.7) | | 3,179(11.9) | 1,084(10.1) |  |
| Month |  |  | |  |  | <0.001 |
| May | 1,658(3.7) | 316(4.4) | | 903(3.4) | 439(4.1) |  |
| June | 2,997(6.7) | 516(7.1) | | 1,769(6.6) | 712(6.7) |  |
| July | 18,289(41.0) | 2,855(39.6) | | 11,107(41.5) | 4,327(40.4) |  |
| August | 18,960(42.5) | 3,132(43.4) | | 11,378(42.6) | 4,450(41.6) |  |
| September | 2,751(6.2) | 398(5.5) | | 1,581(5.9) | 772(7.2) |  |
| **Severity** |  |  | |  |  |  |
| Japan Coma Scale category at admission |  |  | |  |  | <0.001 |
| Alert | 33,488(75.0) | 5,337(74.0) | | 19,956(74.6) | 8,195(76.6) |  |
| Dizzy | 7,005(15.7) | 1,235(17.1) | | 4,217(15.8) | 1,553(14.5) |  |
| Drowsy | 2,047(4.6) | 323(4.5) | | 1,228(4.6) | 496(4.6) |  |
| Come | 2,115(4.7) | 322(4.5) | | 1,337(5.0) | 456(4.3) |  |
| Acute liver failure | 1,281(2.9) | 198(2.7) | | 744(2.8) | 339(3.2) | 0.10 |
| Acute kidney injury | 6,081(13.6) | 849(11.8) | | 3,551(13.3) | 1,681(15.7) | <0.001 |
| Disseminated intravascular coagulation | 954(2.1) | 157(2.2) | | 576(2.2) | 221(2.1) | 0.84 |
| **Intervention** |  |  | |  |  |  |
| Intensive care unit | 1,934(4.3) | 188(2.6) | | 1,343(5.0) | 403(3.8) | <0.001 |
| High care unit | 4,049(9.1) | 682(9.4) | | 2,592(9.7) | 775(7.2) | <0.001 |
| Ambulance use | 27,968(62.6) | 4,446(61.6) | | 17,163(64.2) | 6,359(59.4) | <0.001 |
| Catecholamine use | 1,124(2.5) | 191(2.6) | | 707(2.6) | 226(2.1) | 0.009 |
| Mechanical  ventilation | 1,248(2.8) | 174(2.4) | | 831(3.1) | 243(2.3) | <0.001 |
| Renal replacement therapy | 534(1.2) | 77(1.1) | | 319(1.2) | 138(1.3) | 0.40 |
| **Outcomes** |  |  | |  |  |  |
| In-hospital mortality | 623(1.4) | 118(1.6) | | 375(1.4) | 130(1.2) | 0.062 |
| Length of hospital stay, median (IQR) | 3(2-6) | 3(2-5) | | 3(2-6) | 3(2-5) | <0.001 |
| Hospitalization cost, median (IQR) | 1008(703-1791) | 994(703.-1716) | | 1048(718-1870) | 937  (671-1648) | <0.001 |

Abbreviations; WBGT, wet bulb globe temperature; BMI, body mass index; IQR, interquartile range; AIDS/HIV, acquired immunodeficiency syndrome/human immunodeficiency virus. Data are presented as numbers (%) unless otherwise indicated.

- Long-term local WBGT: The long-term average daily maximum WBGT for each prefecture during the study period.
- Local WBGT areas: We divided the 47 prefectures into three areas using the first and third quartiles of long-term local WBGT (low-WBGT area, 20.79-25.15 °C; middle-WBGT area, 25.16-26.43 °C; and high-WBGT area, 26.44-29.26 °C).

**Table S3** Baseline characteristics of male patients

|  | **Total** | **Low-WBGT**  **20.9-25.1 ℃** | | **Middle-WBGT**  **25.2-26.4 ℃** | **High-WBGT**  **26.5-29.2 ℃** | **p-value** |
| --- | --- | --- | --- | --- | --- | --- |
|  | N=52,363 | N=8,779 | | N=31,433 | N=12,151 |  |
| **Personal-level variables** | |  | |  |  |  |
| Age | 58.5(24.7) | 59.4(25.6) | | 58.7(24.6) | 57.4(24.2) | <0.001 |
| Age category |  |  | |  |  | <0.001 |
| <7 | 569(1.1) | 96(1.1) | | 395(1.3) | 78(0.6) |  |
| 7-17 | 4,605(8.8) | 861(9.8) | | 2,630(8.4) | 1,114(9.2) |  |
| 18-64 | 20,823(39.8) | 3,196(36.4) | | 12,351(39.3) | 5,276(43.4) |  |
| 64-79 | 13,344(25.5) | 2,122(24.2) | | 8,288(26.4) | 2,934(24.1) |  |
| 80 ≤ | 13,022(24.9) | 2,504(28.5) | | 7,769(24.7) | 2,749(22.6) |  |
| BMI category(kg/m^2^) |  |  | |  |  | <0.001 |
| <18.5 | 6,604(12.6) | 1,064(12.1) | | 4,123(13.1) | 1,417(11.7) |  |
| 18.5 – 24.9 | 26,735(51.1) | 4,496(51.2) | | 15,841(50.4) | 6,398(52.7) |  |
| 25.0 – 29.9 | 8,607(16.4) | 1,402(16.0) | | 5,069(16.1) | 2,136(17.6) |  |
| 30.0 ≤ | 2,117(4.0) | 318(3.6) | | 1,277(4.1) | 522(4.3) |  |
| Missing | 8,300(15.9) | 1,499(17.1) | | 5,123(16.3) | 1,678(13.8) |  |
| Smoking | 19,286(36.8) | 3,117(35.5) | | 11,459(36.5) | 4,710(38.8) | <0.001 |
| Missing | 7,668(14.6) | 1,362(15.5) | | 4,817(15.3) | 1,489(12.3) |  |
| Charlson Comorbidity Index | 0(0-1) | 0(0-1) | | 0(0-1) | 0(0-1) | <0.001 |
| Myocardial infarction | 602(1.1) | 105(1.2) | | 370(1.2) | 127(1.0) | 0.46 |
| Congestive  heart failure | 2,014(3.8) | 367(4.2) | | 1,217(3.9) | 430(3.5) | 0.055 |
| Peripheral  vascular disease | 445(0.8) | 87(1.0) | | 272(0.9) | 86(0.7) | 0.079 |
| Cerebrovascular disease | 3,439(6.6) | 618(7.0) | | 2,026(6.4) | 795(6.5) | 0.14 |
| Dementia | 2,280(4.4) | 438(5.0) | | 1,387(4.4) | 455(3.7) | <0.001 |
| Chronic  Pulmonary  disease | 1,605(3.1) | 280(3.2) | | 1,002(3.2) | 323(2.7) | 0.012 |
| Rheumatic disease | 186(0.4) | 30(0.3) | | 102(0.3) | 54(0.4) | 0.16 |
| Peptic ulcer disease | 930(1.8) | | 179(2.0) | 589(1.9) | 162(1.3) | <0.001 |
| Mild liver disease | 2,273(4.3) | 333(3.8) | | 1,314(4.2) | 626(5.2) | <0.001 |
| Diabetes without chronic complication | 4,995(9.5) | 800(9.1) | | 3,037(9.7) | 1,158(9.5) | 0.30 |
| Diabetes with chronic complication | 1,259(2.4) | 209(2.4) | | 773(2.5) | 277(2.3) | 0.54 |
| Hemiplegia/paraplegia | 118(0.2) | 19(0.2) | | 72(0.2) | 27(0.2) | 0.97 |
| Renal disease | 1,348(2.6) | 237(2.7) | | 802(2.6) | 309(2.5) | 0.72 |
| Malignancy | 1,975(3.8) | 386(4.4) | | 1,201(3.8) | 388(3.2) | <0.001 |
| Moderate or severe liver disease | 113(0.2) | 16(0.2) | | 63(0.2) | 34(0.3) | 0.21 |
| Metastatic solid tumor | 300(0.6) | 82(0.9) | | 171(0.5) | 47(0.4) | <0.001 |
| AIDS/HIV | 9(0.0) | 0(0.0) | | 9(0.0) | 0(0.0) | 0.050 |
| Mental disorder | 2,835(5.4) | 470(5.4) | | 1,730(5.5) | 635(5.2) | 0.50 |
| **Admission year and month** | |  | |  |  |  |
| Fiscal year |  |  | |  |  | <0.001 |
| 2011 | 4,481(8.6) | 705(8.0) | | 2,782(8.9) | 994(8.2) |  |
| 2012 | 4,332(8.3) | 827(9.4) | | 2,525(8.0) | 980(8.1) |  |
| 2013 | 5,748(11.0) | 735(8.4) | | 3,539(11.3) | 1,474(12.1) |  |
| 2014 | 4,337(8.3) | 769(8.8) | | 2,632(8.4) | 936(7.7) |  |
| 2015 | 5,754(11.0) | 985(11.2) | | 3,478(11.1) | 1,291(10.6) |  |
| 2016 | 5,964(11.4) | 940(10.7) | | 3,310(10.5) | 1,714(14.1) |  |
| 2017 | 5,535(10.6) | 921(10.5) | | 3,161(10.1) | 1,453(12.0) |  |
| 2018 | 9,505(18.2) | 1,561(17.8) | | 5,960(19.0) | 1,984(16.3) |  |
| 2019 | 6,707(12.8) | 1,336(15.2) | | 4,046(12.9) | 1,325(10.9) |  |
| Month |  |  | |  |  | <0.001 |
| May | 1,861(3.6) | 380(4.3) | | 996(3.2) | 485(4.0) |  |
| June | 3,513(6.7) | 667(7.6) | | 2,049(6.5) | 797(6.6) |  |
| July | 21,454(41.0) | 3,413(38.9) | | 13,139(41.8) | 4,902(40.3) |  |
| August | 22,457(42.9) | 3,839(43.7) | | 13,474(42.9) | 5,144(42.3) |  |
| September | 3,078(5.9) | 480(5.5) | | 1,775(5.6) | 823(6.8) |  |
| **Severity** |  |  | |  |  |  |
| Japan Coma Scale category at admission |  |  | |  |  | <0.001 |
| Alert | 36,894(70.5) | 6,026(68.6) | | 22,015(70.0) | 8,853(72.9) |  |
| Dizzy | 10,399(19.9) | 1,897(21.6) | | 6,241(19.9) | 2,261(18.6) |  |
| Drowsy | 2,601(5.0) | 438(5.0) | | 1,615(5.1) | 548(4.5) |  |
| Come | 2,469(4.7) | 418(4.8) | | 1,562(5.0) | 489(4.0) |  |
| Acute liver failure | 1,401(2.7) | 220(2.5) | | 823(2.6) | 358(2.9) | 0.091 |
| Acute kidney injury | 6,892(13.2) | 981(11.2) | | 4,031(12.8) | 1,880(15.5) | <0.001 |
| Disseminated intravascular coagulation | 1,119(2.1) | 202(2.3) | | 663(2.1) | 254(2.1) | 0.50 |
| **Intervention** |  |  | |  |  |  |
| Intensive care unit | 2,283(4.4) | 227(2.6) | | 1,621(5.2) | 435(3.6) | <0.001 |
| High care unit | 5,190(9.9) | 963(11.0) | | 3,277(10.4) | 950(7.8) | <0.001 |
| Ambulance use | 34,732(66.3) | 5,735(65.3) | | 21,367(68.0) | 7,630(62.8) | <0.001 |
| Catecholamine use | 1,538(2.9) | 279(3.2) | | 941(3.0) | 318(2.6) | 0.039 |
| Mechanical  ventilation | 1,483(2.8) | 231(2.6) | | 975(3.1) | 277(2.3) | <0.001 |
| Renal replacement therapy | 659(1.3) | 107(1.2) | | 393(1.3) | 159(1.3) | 0.83 |
| **Outcomes** |  |  | |  |  |  |
| In-hospital mortality | 1,187(2.3) | 253(2.9) | | 698(2.2) | 236(1.9) | <0.001 |
| Length of hospital stay, median (IQR) | 4(2-8) | 4(2-8) | | 4(2-8) | 3(2-8) | <0.001 |
| Hospitalization cost, median (IQR) | 1236(782-2508) | 1239(784-2540) | | 1286(806-2639) | 1107(732-2214) | <0.001 |

Abbreviations; WBGT, wet bulb globe temperature; BMI, body mass index; IQR, interquartile range; AIDS/HIV, acquired immunodeficiency syndrome/human immunodeficiency virus. Data are presented as numbers (%) unless otherwise indicated.

- Long-term local WBGT: The long-term average daily maximum WBGT for each prefecture during the study period.
- Local WBGT areas: We divided the 47 prefectures into three areas using the first and third quartiles of long-term local WBGT (low-WBGT area, 20.79-25.15 °C; middle-WBGT area, 25.16-26.43 °C; and high-WBGT area, 26.44-29.26 °C).

**Table S4** Baseline characteristics of female patients

|  | **Total** | **Low-WBGT**  **20.9-25.1 ℃** | | **Middle-WBGT**  **25.2-26.4 ℃** | **High-WBGT**  **26.5-29.2 ℃** | **p-value** |
| --- | --- | --- | --- | --- | --- | --- |
|  | **N=29,887** | **N=5,612** | | **N=17,891** | **N=6,384** |  |
| **Personal-level variables** | |  | |  |  |  |
| Age, average (standard deviation) | 71.3(23.5) | 72.7(23.1) | | 71.3(23.5) | 70.4(23.7) | <0.001 |
| Age category |  |  | |  |  | <0.001 |
| <7 | 389(1.3) | 68(1.2) | | 267(1.5) | 54(0.8) |  |
| 7-17 | 2,051(6.9) | 362(6.5) | | 1,165(6.5) | 524(8.2) |  |
| 18-64 | 4,183(14.0) | 712(12.7) | | 2,539(14.2) | 932(14.6) |  |
| 64-79 | 7,882(26.4) | 1,374(24.5) | | 4,765(26.6) | 1,743(27.3) |  |
| 80 ≤ | 15,382(51.5) | 3,096(55.2) | | 9,155(51.2) | 3,131(49.0) |  |
| BMI category(kg/m^2^) |  |  | |  |  | <0.001 |
| <18.5 | 6,382(21.4) | 1,129(20.1) | | 3,943(22.0) | 1,310(20.5) |  |
| 18.5 – 24.9 | 14,264(47.7) | 2,677(47.7) | | 8,395(46.9) | 3,192(50.0) |  |
| 25.0 – 29.9 | 3,182(10.6) | 633(11.3) | | 1,839(10.3) | 710(11.1) |  |
| 30.0 ≤ | 830(2.8) | 164(2.9) | | 478(2.7) | 188(2.9) |  |
| Missing | 5,229(17.5) | 1,009(18.0) | | 3,236(18.1) | 984(15.4) |  |
| Smoking | 25,382(84.9) | 4,855(86.5) | | 15,049(84.1) | 5,478(85.8) | <0.001 |
| Missing | 1,667(5.6) | 262(4.7) | | 1,040(5.8) | 365(5.7) |  |
| Charlson Comorbidity Index | 0(0-1) | 0(0-1) | | 0(0-1) | 0(0-1) | <0.001 |
| Myocardial infarction | 207(0.7) | 31(0.6) | | 129(0.7) | 47(0.7) | 0.37 |
| Congestive  heart failure | 1,901(6.4) | 409(7.3) | | 1,080(6.0) | 412(6.5) | 0.003 |
| Peripheral  vascular disease | 225(0.8) | 41(0.7) | | 131(0.7) | 53(0.8) | 0.72 |
| Cerebrovascular disease | 2,170(7.3) | 431(7.7) | | 1,254(7.0) | 485(7.6) | 0.12 |
| Dementia | 3,041(10.2) | 635(11.3) | | 1,809(10.1) | 597(9.4) | 0.002 |
| Chronic  Pulmonary  disease | 838(2.8) | 145(2.6) | | 497(2.8) | 196(3.1) | 0.26 |
| Rheumatic disease | 441(1.5) | 86(1.5) | | 246(1.4) | 109(1.7) | 0.15 |
| Peptic ulcer disease | 700(2.3) | | 171(3.0) | 411(2.3) | 118(1.8) | <0.001 |
| Mild liver disease | 783(2.6) | 129(2.3) | | 460(2.6) | 194(3.0) | 0.033 |
| Diabetes without chronic complication | 2,786(9.3) | 549(9.8) | | 1,625(9.1) | 612(9.6) | 0.21 |
| Diabetes with chronic complication | 544(1.8) | 91(1.6) | | 338(1.9) | 115(1.8) | 0.42 |
| Hemiplegia/paraplegia | 45(0.2) | 13(0.2) | | 25(0.1) | 7(0.1) | 0.19 |
| Renal disease | 600(2.0) | 103(1.8) | | 357(2.0) | 140(2.2) | 0.37 |
| Malignancy | 920(3.1) | 218(3.9) | | 523(2.9) | 179(2.8) | <0.001 |
| Moderate or severe liver disease | 63(0.2) | 8(0.1) | | 43(0.2) | 12(0.2) | 0.34 |
| Metastatic solid tumor | 157(0.5) | 29(0.5) | | 94(0.5) | 34(0.5) | 0.99 |
| AIDS/HIV | 0(0.0) | 0(0.0) | | 0(0.0) | 0(0.0) |  |
| Mental disorder | 2,506(8.4) | 474(8.4) | | 1,489(8.3) | 543(8.5) | 0.89 |
| **Admission year and month** | |  | |  |  |  |
| Fiscal year |  |  | |  |  | <0.001 |
| 2011 | 2,303(7.7) | 425(7.6) | | 1,414(7.9) | 464(7.3) |  |
| 2012 | 2,175(7.3) | 439(7.8) | | 1,285(7.2) | 451(7.1) |  |
| 2013 | 3,075(10.3) | 379(6.8) | | 1,940(10.8) | 756(11.8) |  |
| 2014 | 2,116(7.1) | 428(7.6) | | 1,269(7.1) | 419(6.6) |  |
| 2015 | 3,562(11.9) | 691(12.3) | | 2,099(11.7) | 772(12.1) |  |
| 2016 | 3,295(11.0) | 560(10.0) | | 1,828(10.2) | 907(14.2) |  |
| 2017 | 3,072(10.3) | 597(10.6) | | 1,724(9.6) | 751(11.8) |  |
| 2018 | 6,212(20.8) | 1,166(20.8) | | 3,916(21.9) | 1,130(17.7) |  |
| 2019 | 4,077(13.6) | 927(16.5) | | 2,416(13.5) | 734(11.5) |  |
| Month |  |  | |  |  | <0.001 |
| May | 910(3.0) | 217(3.9) | | 492(2.7) | 201(3.1) |  |
| June | 1,723(5.8) | 283(5.0) | | 1,087(6.1) | 353(5.5) |  |
| July | 13,379(44.8) | 2,376(42.3) | | 8,085(45.2) | 2,918(45.7) |  |
| August | 12,575(42.1) | 2,529(45.1) | | 7,451(41.6) | 2,595(40.6) |  |
| September | 1,300(4.3) | 207(3.7) | | 776(4.3) | 317(5.0) |  |
| **Severity** |  |  | |  |  |  |
| Japan Coma Scale category at admission |  |  | |  |  | <0.001 |
| Alert | 19,496(65.2) | 3,516(62.7) | | 11,655(65.1) | 4,325(67.7) |  |
| Dizzy | 7,009(23.5) | 1,433(25.5) | | 4,197(23.5) | 1,379(21.6) |  |
| Drowsy | 1,873(6.3) | 354(6.3) | | 1,114(6.2) | 405(6.3) |  |
| Come | 1,509(5.0) | 309(5.5) | | 925(5.2) | 275(4.3) |  |
| Acute liver failure | 433(1.4) | 62(1.1) | | 267(1.5) | 104(1.6) | 0.042 |
| Acute kidney injury | 1,044(3.5) | 170(3.0) | | 632(3.5) | 242(3.8) | 0.069 |
| Disseminated intravascular coagulation | 588(2.0) | 111(2.0) | | 359(2.0) | 118(1.8) | 0.74 |
| **Intervention** |  |  | |  |  |  |
| Intensive care unit | 1,184(4.0) | 126(2.2) | | 842(4.7) | 216(3.4) | <0.001 |
| High care unit | 2,588(8.7) | 492(8.8) | | 1,636(9.1) | 460(7.2) | <0.001 |
| Ambulance use | 19,700(65.9) | 3,548(63.2) | | 11,967(66.9) | 4,185(65.6) | <0.001 |
| Catecholamine use | 978(3.3) | 190(3.4) | | 601(3.4) | 187(2.9) | 0.22 |
| Mechanical  ventilation | 774(2.6) | 127(2.3) | | 507(2.8) | 140(2.2) | 0.005 |
| Renal replacement therapy | 217(0.7) | 26(0.5) | | 133(0.7) | 58(0.9) | 0.015 |
| **Outcomes** |  |  | |  |  |  |
| In-hospital mortality | 857(2.9) | 203(3.6) | | 496(2.8) | 158(2.5) | <0.001 |
| Length of hospital stay, median (IQR) | 6(3-13) | 6(3-14) | | 5(3-13) | 6(3-14) | 0.14 |
| Hospitalization cost, median (IQR) | 1640(906-347) | 1653 (928-3498) | | 1656(918-3523) | 1577(853-3423) | <0.001 |

Abbreviations; WBGT, wet bulb globe temperature; BMI, body mass index; IQR, interquartile range; AIDS/HIV, acquired immunodeficiency syndrome/human immunodeficiency virus. Data are presented as numbers (%) unless otherwise indicated.

- Long-term local WBGT: The long-term average daily maximum WBGT for each prefecture during the study period.
- Local WBGT areas: We divided the 47 prefectures into three areas using the first and third quartiles of long-term local WBGT (low-WBGT area, 20.79-25.15 °C; middle-WBGT area, 25.16-26.43 °C; and high-WBGT area, 26.44-29.26 °C).

**Table S5** Baseline characteristics of underweight patients

|  | **Total** | **Low-WBGT**  **20.9-25.1 ℃** | | **Middle-WBGT**  **25.2-26.4 ℃** | **High-WBGT**  **26.5-29.2 ℃** | **p-value** |
| --- | --- | --- | --- | --- | --- | --- |
|  | **N=12,986** | **N=2,193** | | **N=8,066** | **N=2,727** |  |
| **Personal-level variables** | |  | |  |  |  |
| Age, average (standard deviation) | 64.0 (30.2) | 65.8(30.2) | | 63.4(30.5) | 64.3(29.3) | 0.003 |
| Age category |  |  | |  |  | <0.001 |
| <7 | 672(5.2) | 112(5.1) | | 481(6.0) | 79(2.9) |  |
| 7-17 | 1,843(14.2) | 288(13.1) | | 1,133(14.0) | 422(15.5) |  |
| 18-64 | 1,611(12.4) | 234(10.7) | | 992(12.3) | 385(14.1) |  |
| 64-79 | 2,820(21.7) | 436(19.9) | | 1,795(22.3) | 589(21.6) |  |
| 80 ≤ | 6,040(46.5) | 1,123(51.2) | | 3,665(45.4) | 1,252(45.9) |  |
| Male | 6,604(50.9) | 1,064(48.5) | | 4,123(51.1) | 1,417(52.0) | 0.042 |
| BMI (kg/m^2^) | 16.6143(1.516928) | 16.66111(1.493166) | | 16.5665(1.537592) | 16.71806(1.467734) | <0.001 |
| Smoking | 2,412(18.6) | 370(16.9) | | 1,505(18.7) | 537(19.7) |  |
| Missing | 1,216(9.4) | 188(8.6) | | 792(9.8) | 236(8.7) |  |
| Charlson Comorbidity Index | 0(0-1) | 0(0-1) | | 0(0-1) | 0(0-1) | 0.094 |
| Myocardial infarction | 109(0.8) | 18(0.8) | | 71(0.9) | 20(0.7) | 0.76 |
| Congestive  heart failure | 771(5.9) | 142(6.5) | | 478(5.9) | 151(5.5) | 0.38 |
| Peripheral  vascular disease | 117(0.9) | 20(0.9) | | 73(0.9) | 24(0.9) | 0.99 |
| Cerebrovascular disease | 928(7.1) | 168(7.7) | | 555(6.9) | 205(7.5) | 0.32 |
| Dementia | 1,250(9.6) | 231(10.5) | | 775(9.6) | 244(8.9) | 0.17 |
| Chronic  Pulmonary  disease | 620(4.8) | 106(4.8) | | 387(4.8) | 127(4.7) | 0.95 |
| Rheumatic disease | 147(1.1) | 24(1.1) | | 90(1.1) | 33(1.2) | 0.91 |
| Peptic ulcer disease | 295(2.3) | | 68(3.1) | 184(2.3) | 43(1.6) | 0.002 |
| Mild liver disease | 380(2.9) | 44(2.0) | | 245(3.0) | 91(3.3) | 0.014 |
| Diabetes without chronic complication | 786(6.1) | 138(6.3) | | 485(6.0) | 163(6.0) | 0.87 |
| Diabetes with chronic complication | 218(1.7) | 40(1.8) | | 122(1.5) | 56(2.1) | 0.14 |
| Hemiplegia/paraplegia | 24(0.2) | 5(0.2) | | 16(0.2) | 3(0.1) | 0.57 |
| Renal disease | 330(2.5) | 56(2.6) | | 202(2.5) | 72(2.6) | 0.93 |
| Malignancy | 642(4.9) | 125(5.7) | | 397(4.9) | 120(4.4) | 0.11 |
| Moderate or severe liver disease | 18(0.1) | 3(0.1) | | 7(0.1) | 8(0.3) | 0.043 |
| Metastatic solid tumor | 116(0.9) | 25(1.1) | | 75(0.9) | 16(0.6) | 0.10 |
| AIDS/HIV | 3(0.0) | 0(0.0) | | 3(0.0) | 0(0.0) | 0.40 |
| Mental disorder | 932(7.2) | 154(7.0) | | 588(7.3) | 190(7.0) | 0.81 |
| **Admission year and month** | |  | |  |  |  |
| Fiscal year |  |  | |  |  | <0.001 |
| 2011 | 985(7.6) | 146(6.7) | | 646(8.0) | 193(7.1) |  |
| 2012 | 970(7.5) | 167(7.6) | | 598(7.4) | 205(7.5) |  |
| 2013 | 1,400(10.8) | 156(7.1) | | 940(11.7) | 304(11.1) |  |
| 2014 | 951(7.3) | 173(7.9) | | 610(7.6) | 168(6.2) |  |
| 2015 | 1,570(12.1) | 269(12.3) | | 977(12.1) | 324(11.9) |  |
| 2016 | 1,405(10.8) | 208(9.5) | | 810(10.0) | 387(14.2) |  |
| 2017 | 1,333(10.3) | 226(10.3) | | 799(9.9) | 308(11.3) |  |
| 2018 | 2,584(19.9) | 442(20.2) | | 1,647(20.4) | 495(18.2) |  |
| 2019 | 1,788(13.8) | 406(18.5) | | 1,039(12.9) | 343(12.6) |  |
| Month |  |  | |  |  | <0.001 |
| May | 439(3.4) | 87(4.0) | | 253(3.1) | 99(3.6) |  |
| June | 833(6.4) | 136(6.2) | | 527(6.5) | 170(6.2) |  |
| July | 5,554(42.8) | 846(38.6) | | 3,560(44.1) | 1,148(42.1) |  |
| August | 5,539(42.7) | 1,031(47.0) | | 3,337(41.4) | 1,171(42.9) |  |
| September | 621(4.8) | 93(4.2) | | 389(4.8) | 139(5.1) |  |
| **Severity** |  |  | |  |  |  |
| Japan Coma Scale category at admission |  |  | |  |  | 0.95 |
| Alert | 8,540(65.8) | 1,426(65.0) | | 5,314(65.9) | 1,800(66.0) |  |
| Dizzy | 3,021(23.3) | 529(24.1) | | 1,855(23.0) | 637(23.4) |  |
| Drowsy | 825(6.4) | 138(6.3) | | 519(6.4) | 168(6.2) |  |
| Come | 600(4.6) | 100(4.6) | | 378(4.7) | 122(4.5) |  |
| Acute liver failure | 200(1.5) | 24(1.1) | | 131(1.6) | 45(1.7) | 0.18 |
| Acute kidney injury | 670(5.2) | 88(4.0) | | 407(5.0) | 175(6.4) | <0.001 |
| Disseminated intravascular coagulation | 261(2.0) | 35(1.6) | | 182(2.3) | 44(1.6) | 0.037 |
| **Intervention** |  |  | |  |  |  |
| Intensive care unit | 544(4.2) | 35(1.6) | | 408(5.1) | 101(3.7) | <0.001 |
| High care unit | 1,188(9.1) | 181(8.3) | | 777(9.6) | 230(8.4) | 0.048 |
| Ambulance use | 8,116(62.5) | 1,264(57.6) | | 5,165(64.0) | 1,687(61.9) | <0.001 |
| Catecholamine use | 431(3.3) | 69(3.1) | | 280(3.5) | 82(3.0) | 0.45 |
| Mechanical  ventilation | 305(2.3) | 47(2.1) | | 218(2.7) | 40(1.5) | <0.001 |
| Renal replacement therapy | 138(1.1) | 19(0.9) | | 81(1.0) | 38(1.4) | 0.14 |
| **Outcomes** |  |  | |  |  |  |
| In-hospital mortality | 441(3.4) | 100(4.6) | | 267(3.3) | 74(2.7) | 0.001 |
| Length of hospital stay, median (IQR) | 6(3-16) | 6(3-17) | | 6(3-15) | 6(3-16) | 0.36 |
| Hospitalization cost, median (IQR) | 1732 (888-4052) | 1763 (881-4179) | | 1722(897-4045) | 1726.04(860-3982) | 0.64 |

Abbreviations; WBGT, wet bulb globe temperature; BMI, body mass index; IQR, interquartile range; AIDS/HIV, acquired immunodeficiency syndrome/human immunodeficiency virus. Data are presented as numbers (%) unless otherwise indicated.

- Long-term local WBGT: The long-term average daily maximum WBGT for each prefecture during the study period.
- Local WBGT areas: We divided the 47 prefectures into three areas using the first and third quartiles of long-term local WBGT (low-WBGT area, 20.79-25.15 °C; middle-WBGT area, 25.16-26.43 °C; and high-WBGT area, 26.44-29.26 °C).

**Table S6** Baseline characteristics of patients with normal weight

|  | **Total** | **Low-WBGT**  **20.9-25.1 ℃** | | **Middle-WBGT**  **25.2-26.4 ℃** | **High-WBGT**  **26.5-29.2 ℃** | **p-value** |
| --- | --- | --- | --- | --- | --- | --- |
|  | **N=40,956** | **N=7,168** | | **N=24,210** | **N=9,578** |  |
| **Personal-level variables** | |  | |  |  |  |
| Age, average (standard deviation) | 63.9(24.2) | 65.5(24.6) | | 64.1(24.0) | 62.3(24.4) | <0.001 |
| Age category |  |  | |  |  |  |
| <7 | 57(0.1) | 7(0.1) | | 44(0.2) | 6(0.1) | <0.001 |
| 7-17 | 3,238(7.9) | 593(8.3) | | 1,778(7.3) | 867(9.1) |  |
| 18-64 | 12,426(30.3) | 1,960(27.3) | | 7,324(30.3) | 3,142(32.8) |  |
| 64-79 | 11,080(27.1) | 1,778(24.8) | | 6,756(27.9) | 2,546(26.6) |  |
| 80 ≤ | 14,155(34.6) | 2,830(39.5) | | 8,308(34.3) | 3,017(31.5) |  |
| Male | 26,703(65.2) | 4,494(62.7) | | 15,821(65.3) | 6,388(66.7) | <0.001 |
| BMI (kg/m^2^) | 21.7(1. 8) | 21.7(1.8) | | 21.7 (1.8) | 21.7 (1.8) | 0.83 |
| Smoking | 11,271(27.5) | 1,859(25.9) | | 6,643(27.4) | 2,769(28.9) | <0.001 |
| Missing | 4,223(10.3) | 771(10.8) | | 2,621(10.8) | 831(8.7) |  |
| Charlson Comorbidity Index | 0(0-1) | 0(0-1) | | 0(0-1) | 0(0-1) | <0.001 |
| Myocardial infarction | 433(1.1) | 81(1.1) | | 252(1.0) | 100(1.0) | 0.8 |
| Congestive  heart failure | 2,028(5.0) | 393(5.5) | | 1,183(4.9) | 452(4.7) | 0.06 |
| Peripheral  vascular disease | 359(0.9) | 76(1.1) | | 209(0.9) | 74(0.8) | 0.13 |
| Cerebrovascular disease | 2,935(7.2) | 556(7.8) | | 1,680(6.9) | 699(7.3) | 0.053 |
| Dementia | 2,587(6.3) | 536(7.5) | | 1,536(6.3) | 515(5.4) | <0.001 |
| Chronic  Pulmonary  disease | 1,134(2.8) | 186(2.6) | | 697(2.9) | 251(2.6) | 0.26 |
| Rheumatic disease | 321(0.8) | 60(0.8) | | 167(0.7) | 94(1.0) | 0.02 |
| Peptic ulcer disease | 866(2.1) | | 201(2.8) | 529(2.2) | 136(1.4) | <0.001 |
| Mild liver disease | 1,567(3.8) | 248(3.5) | | 916(3.8) | 403(4.2) | 0.038 |
| Diabetes without chronic complication | 3,912(9.6) | 697(9.7) | | 2,291(9.5) | 924(9.6) | 0.75 |
| Diabetes with chronic complication | 959(2.3) | 166(2.3) | | 584(2.4) | 209(2.2) | 0.45 |
| Hemiplegia/paraplegia | 82(0.2) | 18(0.3) | | 46(0.2) | 18(0.2) | 0.57 |
| Renal disease | 1,063(2.6) | 171(2.4) | | 621(2.6) | 271(2.8) | 0.18 |
| Malignancy | 1,552(3.8) | 337(4.7) | | 905(3.7) | 310(3.2) | <0.001 |
| Moderate or severe liver disease | 88(0.2) | 12(0.2) | | 51(0.2) | 25(0.3) | 0.42 |
| Metastatic solid tumor | 253(0.6) | 66(0.9) | | 138(0.6) | 49(0.5) | 0.001 |
| AIDS/HIV | 6(0.0) | 0(0.0) | | 6(0.0) | 0(0.0) | 0.13 |
| Mental disorder | 2,577(6.3) | 467(6.5) | | 1,517(6.3) | 593(6.2) | 0.67 |
| **Admission year and month** | |  | |  |  |  |
| Fiscal year |  |  | |  |  | <0.001 |
| 2011 | 3,230(7.9) | 538(7.5) | | 1,962(8.1) | 730(7.6) |  |
| 2012 | 3,232(7.9) | 622(8.7) | | 1,858(7.7) | 752(7.9) |  |
| 2013 | 4,280(10.5) | 552(7.7) | | 2,601(10.7) | 1,127(11.8) |  |
| 2014 | 3,268(8.0) | 629(8.8) | | 1,925(8.0) | 714(7.5) |  |
| 2015 | 4,654(11.4) | 841(11.7) | | 2,752(11.4) | 1,061(11.1) |  |
| 2016 | 4,736(11.6) | 751(10.5) | | 2,593(10.7) | 1,392(14.5) |  |
| 2017 | 4,333(10.6) | 723(10.1) | | 2,449(10.1) | 1,161(12.1) |  |
| 2018 | 7,921(19.3) | 1,400(19.5) | | 4,899(20.2) | 1,622(16.9) |  |
| 2019 | 5,302(12.9) | 1,112(15.5) | | 3,171(13.1) | 1,019(10.6) |  |
| Month |  |  | |  |  | <0.001 |
| May | 1,379(3.4) | 300(4.2) | | 720(3.0) | 359(3.7) |  |
| June | 2,590(6.3) | 482(6.7) | | 1,534(6.3) | 574(6.0) |  |
| July | 17,525(42.8) | 2,918(40.7) | | 10,531(43.5) | 4,076(42.6) |  |
| August | 17,325(42.3) | 3,143(43.8) | | 10,183(42.1) | 3,999(41.8) |  |
| September | 2,137(5.2) | 325(4.5) | | 1,242(5.1) | 570(6.0) |  |
| **Severity** |  |  | |  |  |  |
| Japan Coma Scale category at  admission |  |  | |  |  | <0.001 |
| Alert | 28,619(69.9) | 4,845(67.6) | | 16,862(69.6) | 6,912(72.2) |  |
| Dizzy | 8,449(20.6) | 1,624(22.7) | | 4,977(20.6) | 1,848(19.3) |  |
| Drowsy | 2,150(5.2) | 385(5.4) | | 1,281(5.3) | 484(5.1) |  |
| Come | 1,738(4.2) | 314(4.4) | | 1,090(4.5) | 334(3.5) |  |
| Acute liver failure | 900(2.2) | 140(2.0) | | 534(2.2) | 226(2.4) | 0.2 |
| Acute kidney injury | 3,937(9.6) | 556(7.8) | | 2,275(9.4) | 1,106(11.5) | <0.001 |
| Disseminated intravascular coagulation | 825(2.0) | 156(2.2) | | 481(2.0) | 188(2.0) | 0.56 |
| **Intervention** |  |  | |  |  |  |
| Intensive care unit | 1,699(4.1) | 170(2.4) | | 1,217(5.0) | 312(3.3) | <0.001 |
| High care unit | 3,842(9.4) | 744(10.4) | | 2,356(9.7) | 742(7.7) | <0.001 |
| Ambulance use | 26,760(65.3) | 4,584(64.0) | | 16,162(66.8) | 6,014(62.8) | <0.001 |
| Catecholamine use | 1,085(2.6) | 207(2.9) | | 652(2.7) | 226(2.4) | 0.087 |
| Mechanical  ventilation | 989(2.4) | 155(2.2) | | 652(2.7) | 182(1.9) | <0.001 |
| Renal replacement  therapy | 437(1.1) | 73(1.0) | | 246(1.0) | 118(1.2) | 0.2 |
| **Outcomes** |  |  | |  |  |  |
| In-hospital mortality | 695(1.7) | 165(2.3) | | 396(1.6) | 134(1.4) | <0.001 |
| Length of hospital stay, median (IQR) | 4(2-10) | 4(2-10) | | 4(2-10) | 4(2-9) | <0.001 |
| Hospitalization cost,  median (IQR) | 1369(834-2815) | 1409(864-2927) | | 1410(853-2894) | 1237(771-2573) | <0.001 |

Abbreviations; WBGT, wet bulb globe temperature; BMI, body mass index; IQR, interquartile range; AIDS/HIV, acquired immunodeficiency syndrome/human immunodeficiency virus. Data are presented as numbers (%) unless otherwise indicated.

- Long-term local WBGT: The long-term average daily maximum WBGT for each prefecture during the study period.
- Local WBGT areas: We divided the 47 prefectures into three areas using the first and third quartiles of long-term local WBGT (low-WBGT area, 20.7925.15 °C; middle-WBGT area, 25.16-26.43 °C; and high-WBGT area, 26.44-29.26 °C).

**Table 7** Baseline characteristics of patients with overweight or obesity

|  | **Total** | **Low-WBGT**  **20.9-25.1 ℃** | | **Middle-WBGT**  **25.2-26.4 ℃** | **High-WBGT**  **26.5-29.2 ℃** | **p-value** |
| --- | --- | --- | --- | --- | --- | --- |
|  | **N=14,779** | **N=2,522** | | **N=8,689** | **N=3,568** |  |
| **Personal-level variables** | |  | |  |  |  |
| Age, average (standard deviation) | 60.7(20.6) | 63.0(20.9) | | 60.6(20.4) | 59.4(20.9) | <0.001 |
| Age category |  |  | |  |  |  |
| <7 | 7(0.0) | 2(0.1) | | 4(0.0) | 1(0.0) | <0.001 |
| 7-17 | 395(2.7) | 72(2.9) | | 207(2.4) | 116(3.3) |  |
| 18-64 | 6,959(47.1) | 1,036(41.1) | | 4,134(47.6) | 1,789(50.1) |  |
| 64-79 | 4,234(28.6) | 753(29.9) | | 2,534(29.2) | 947(26.5) |  |
| 80 ≤ | 3,184(21.5) | 659(26.1) | | 1,810(20.8) | 715(20.0) |  |
| Male | 10,756(72.8) | 1,722(68.3) | | 6,366(73.3) | 2,668(74.8) | <0.001 |
| BMI (kg/m^2^) | 28.2(3.2) | 28.1(3.2) | | 28.2(3.2) | 28.2(3.2) | 0.64 |
| Smoking | 5,029(34.0) | 793(31.4) | | 2,932(33.7) | 1,304(36.5) |  |
| Missing | 1,591(10.8) | 303(12.0) | | 974(11.2) | 314(8.8) |  |
| Charlson Comorbidity Index | 0(0-1) | 0(0-1) | | 0(0-1) | 0(0-1) | 0.026 |
| Myocardial infarction | 177(1.2) | 24(1.0) | | 116(1.3) | 37(1.0) | 0.18 |
| Congestive  heart failure | 613(4.1) | 140(5.6) | | 334(3.8) | 139(3.9) | <0.001 |
| Peripheral  vascular disease | 125(0.8) | 20(0.8) | | 75(0.9) | 30(0.8) | 0.94 |
| Cerebrovascular disease | 948(6.4) | 183(7.3) | | 552(6.4) | 213(6.0) | 0.12 |
| Dementia | 528(3.6) | 114(4.5) | | 304(3.5) | 110(3.1) | 0.010 |
| Chronic  Pulmonary  disease | 384(2.6) | 79(3.1) | | 220(2.5) | 85(2.4) | 0.16 |
| Rheumatic disease | 86(0.6) | 16(0.6) | | 48(0.6) | 22(0.6) | 0.85 |
| Peptic ulcer disease | 263(1.8) | | 51(2.0) | 154(1.8) | 58(1.6) | 0.51 |
| Mild liver disease | 724(4.9) | 115(4.6) | | 381(4.4) | 228(6.4) | <0.001 |
| Diabetes without chronic complication | 2,126(14.4) | 358(14.2) | | 1,268(14.6) | 500(14.0) | 0.68 |
| Diabetes with chronic complication | 473(3.2) | 72(2.9) | | 305(3.5) | 96(2.7) | 0.036 |
| Hemiplegia/paraplegia | 30(0.2) | 4(0.2) | | 20(0.2) | 6(0.2) | 0.68 |
| Renal disease | 321(2.2) | 63(2.5) | | 188(2.2) | 70(2.0) | 0.37 |
| Malignancy | 392(2.7) | 75(3.0) | | 238(2.7) | 79(2.2) | 0.14 |
| Moderate or severe liver disease | 49(0.3) | 6(0.2) | | 35(0.4) | 8(0.2) | 0.20 |
| Metastatic solid tumor | 50(0.3) | 10(0.4) | | 30(0.3) | 10(0.3) | 0.73 |
| AIDS/HIV | 0(0.0) | 0(0.0) | | 0(0.0) | 0(0.0) |  |
| Mental disorder | 988(6.7) | 184(7.3) | | 572(6.6) | 232(6.5) | 0.40 |
| Admission year and month | |  | |  |  |  |
| Fiscal year |  |  | |  |  | <0.001 |
| 2011 | 1,118(7.6) | 172(6.8) | | 682(7.8) | 264(7.4) |  |
| 2012 | 1,110(7.5) | 226(9.0) | | 637(7.3) | 247(6.9) |  |
| 2013 | 1,536(10.4) | 194(7.7) | | 929(10.7) | 413(11.6) |  |
| 2014 | 1,207(8.2) | 216(8.6) | | 719(8.3) | 272(7.6) |  |
| 2015 | 1,601(10.8) | 284(11.3) | | 946(10.9) | 371(10.4) |  |
| 2016 | 1,756(11.9) | 290(11.5) | | 941(10.8) | 525(14.7) |  |
| 2017 | 1,629(11.0) | 290(11.5) | | 889(10.2) | 450(12.6) |  |
| 2018 | 2,829(19.1) | 472(18.7) | | 1,761(20.3) | 596(16.7) |  |
| 2019 | 1,993(13.5) | 378(15.0) | | 1,185(13.6) | 430(12.1) |  |
| Month |  |  | |  |  | 0.005 |
| May | 493(3.3) | 104(4.1) | | 264(3.0) | 125(3.5) |  |
| June | 914(6.2) | 153(6.1) | | 532(6.1) | 229(6.4) |  |
| July | 5,991(40.5) | 1,018(40.4) | | 3,536(40.7) | 1,437(40.3) |  |
| August | 6,504(44.0) | 1,099(43.6) | | 3,882(44.7) | 1,523(42.7) |  |
| September | 877(5.9) | 148(5.9) | | 475(5.5) | 254(7.1) |  |
| **Severity** |  |  | |  |  |  |
| Japan Coma Scale category at  admission |  |  | |  |  | <0.001 |
| Alert | 10,658(72.1) | 1,713(67.9) | | 6,288(72.4) | 2,657(74.5) |  |
| Dizzy | 2,781(18.8) | 562(22.3) | | 1,602(18.4) | 617(17.3) |  |
| Drowsy | 643(4.4) | 121(4.8) | | 375(4.3) | 147(4.1) |  |
| Come | 697(4.7) | 126(5.0) | | 424(4.9) | 147(4.1) |  |
| Acute liver failure | 454(3.1) | 71(2.8) | | 258(3.0) | 125(3.5) | 0.21 |
| Acute kidney injury | 2,171(14.7) | 298(11.8) | | 1,295(14.9) | 578(16.2) | <0.001 |
| Disseminated intravascular coagulation | 336(2.3) | 65(2.6) | | 199(2.3) | 72(2.0) | 0.35 |
| **Intervention** |  |  | |  |  |  |
| Intensive care unit | 693(4.7) | 91(3.6) | | 461(5.3) | 141(4.0) | <0.001 |
| High care unit | 1,516(10.3) | 315(12.5) | | 919(10.6) | 282(7.9) | <0.001 |
| Ambulance use | 9,647(65.3) | 1,654(65.6) | | 5,772(66.4) | 2,221(62.2) | <0.001 |
| Catecholamine use | 438(3.0) | 83(3.3) | | 265(3.0) | 90(2.5) | 0.17 |
| Mechanical  ventilation | 456(3.1) | 72(2.9) | | 289(3.3) | 95(2.7) | 0.12 |
| Renal replacement  therapy | 197(1.3) | 30(1.2) | | 124(1.4) | 43(1.2) | 0.49 |
| **Outcomes** |  |  | |  |  |  |
| In-hospital mortality | 227(1.5) | 46(1.8) | | 129(1.5) | 52(1.5) | 0.43 |
| Length of hospital stay, median (IQR) | 4(2-8) | 4(2-9) | | 4(2-8) | 4(2-8) | 0.002 |
| Hospitalization cost,  median (IQR) | 1308(823-2572) | 1386(857-2834) | | 1337(846-2605) | 1194(759-2325) | <0.001 |

Abbreviations; WBGT, wet bulb globe temperature; BMI, body mass index; IQR, interquartile range; AIDS/HIV, acquired immunodeficiency syndrome/human immunodeficiency virus. Data are presented as numbers (%) unless otherwise indicated.

- Long-term local WBGT: The long-term average daily maximum WBGT for each prefecture during the study period.
- Local WBGT areas: We divided the 47 prefectures into three areas using the first and third quartiles of long-term local WBGT (low-WBGT area, 20.79-25.15 °C; middle-WBGT area, 25.16-26.43 °C; and high-WBGT area, 26.44-29.26 °C).

**Table 8** Baseline characteristics of patients with no comorbidities (CCI = 0)

|  | **Total** | **Low-WBGT**  **20.9-25.1 ℃** | **Middle-WBGT**  **25.2-26.4 ℃** | **High-WBGT**  **26.5-29.2 ℃** | **p-value** |
| --- | --- | --- | --- | --- | --- |
|  | **N=53,274** | **N=9,093** | **N=32,026** | **N=12,155** |  |
| **Personal-level variables** | |  |  |  |  |
| Age, average (standard deviation) | 56.4(26.8) | 57.2(27.5) | 56.7(26.8) | 55.2(26.3) | <0.001 |
| Age category |  |  |  |  | <0.001 |
| <7 | 901(1.7) | 154(1.7) | 625(2.0) | 122(1.0) |  |
| 7-17 | 6,417(12.0) | 1,164(12.8) | 3,664(11.4) | 1,589(13.1) |  |
| 18-64 | 20,350(38.2) | 3,235(35.6) | 12,115(37.8) | 5,000(41.1) |  |
| 64-79 | 11,558(21.7) | 1,870(20.6) | 7,122(22.2) | 2,566(21.1) |  |
| 80 ≤ | 14,048(26.4) | 2,670(29.4) | 8,500(26.5) | 2,878(23.7) |  |
| Male | 34,915(65.5) | 5,777(63.5) | 20,898(65.3) | 8,240(67.8) | <0.001 |
| BMI category(kg/m^2^) |  |  |  |  | <0.001 |
| <18.5 | 8,075(15.2) | 1,322(14.5) | 5,055(15.8) | 1,698(14.0) |  |
| 18.5 - 25.0 | 26,294(49.4) | 4,447(48.9) | 15,548(48.5) | 6,299(51.8) |  |
| 25.0 - 30.0 | 7,488(14.1) | 1,248(13.7) | 4,410(13.8) | 1,830(15.1) |  |
| 30.0 ≤ | 1,851(3.5) | 291(3.2) | 1,111(3.5) | 449(3.7) |  |
| Missing | 9,566(18.0) | 1,785(19.6) | 5,902(18.4) | 1,879(15.5) |  |
| Smoking | 13,274(24.9) | 2,161(23.8) | 7,825(24.4) | 3,288(27.1) | <0.001 |
| Missing | 6,966(13.1) | 1,234(13.6) | 4,403(13.7) | 1,329(10.9) |  |
| Mental disorder | 3,444(6.5) | 593(6.5) | 2,087(6.5) | 764(6.3) | 0.66 |
| **Admission year and month** | |  |  |  |  |
| Fiscal year |  |  |  |  | <0.001 |
| 2011 | 4,522(8.5) | 741(8.1) | 2,802(8.7) | 979(8.1) |  |
| 2012 | 4,553(8.5) | 848(9.3) | 2,683(8.4) | 1,022(8.4) |  |
| 2013 | 5,948(11.2) | 728(8.0) | 3,708(11.6) | 1,512(12.4) |  |
| 2014 | 4,366(8.2) | 768(8.4) | 2,682(8.4) | 916(7.5) |  |
| 2015 | 5,994(11.3) | 1,025(11.3) | 3,623(11.3) | 1,346(11.1) |  |
| 2016 | 5,912(11.1) | 948(10.4) | 3,286(10.3) | 1,678(13.8) |  |
| 2017 | 5,492(10.3) | 923(10.2) | 3,162(9.9) | 1,407(11.6) |  |
| 2018 | 9,812(18.4) | 1,742(19.2) | 6,109(19.1) | 1,961(16.1) |  |
| 2019 | 6,675(12.5) | 1,370(15.1) | 3,971(12.4) | 1,334(11.0) |  |
| Month |  |  |  |  | <0.001 |
| May | 1,940(3.6) | 417(4.6) | 1,050(3.3) | 473(3.9) |  |
| June | 3,573(6.7) | 643(7.1) | 2,138(6.7) | 792(6.5) |  |
| July | 22,171(41.6) | 3,633(40.0) | 13,538(42.3) | 5,000(41.1) |  |
| August | 22,560(42.3) | 3,942(43.4) | 13,510(42.2) | 5,108(42.0) |  |
| September | 3,030(5.7) | 458(5.0) | 1,790(5.6) | 782(6.4) |  |
| **Severity** |  |  |  |  |  |
| Japan Coma Scale category at admission |  |  |  |  | <0.001 |
| Alert | 37,635(70.6) | 6,181(68.0) | 22,559(70.4) | 8,895(73.2) |  |
| Dizzy | 10,177(19.1) | 1,953(21.5) | 6,079(19.0) | 2,145(17.6) |  |
| Drowsy | 2,739(5.1) | 451(5.0) | 1,692(5.3) | 596(4.9) |  |
| Come | 2,723(5.1) | 508(5.6) | 1,696(5.3) | 519(4.3) |  |
| Acute liver failure | 255(0.5) | 45(0.5) | 158(0.5) | 52(0.4) | 0.65 |
| Acute kidney injury | 5,807(10.9) | 852(9.4) | 3,413(10.7) | 1,542(12.7) | <0.001 |
| Disseminated intravascular coagulation | 1,148(2.2) | 218(2.4) | 675(2.1) | 255(2.1) | 0.22 |
| **Intervention** |  |  |  |  |  |
| Intensive care unit | 2,285(4.3) | 235(2.6) | 1,627(5.1) | 423(3.5) | <0.001 |
| High care unit | 5,222(9.8) | 1,008(11.1) | 3,318(10.4) | 896(7.4) | <0.001 |
| Ambulance use | 35,184(66.0) | 5,971(65.7) | 21,562(67.3) | 7,651(62.9) | <0.001 |
| Catecholamine use | 1,478(2.8) | 289(3.2) | 907(2.8) | 282(2.3) | <0.001 |
| Mechanical  ventilation | 1,588(3.0) | 260(2.9) | 1,030(3.2) | 298(2.5) | <0.001 |
| Renal replacement therapy | 374(0.7) | 57(0.6) | 228(0.7) | 89(0.7) | 0.62 |
| **Outcomes** |  |  |  |  |  |
| In-hospital mortality | 1,133(2.1) | 242(2.7) | 673(2.1) | 218(1.8) | <0.001 |
| Length of hospital stay, median (IQR) | 3(2-7) | 3(2-7) | 3(2-7) | 3(2-6) | <0.001 |
| Hospitalization cost, median (IQR) | 1102(735-2119) | 1119(745-2176) | 1140(751-2187) | 995(695-1909) | <0.001 |

Abbreviations; WBGT, wet bulb globe temperature; BMI, body mass index; IQR, interquartile range; AIDS/HIV, acquired immunodeficiency syndrome/human immunodeficiency virus. Data are presented as numbers (%) unless otherwise indicated.

- Long-term local WBGT: The long-term average daily maximum WBGT for each prefecture during the study period.
- Local WBGT areas: We divided the 47 prefectures into three areas using the first and third quartiles of long-term local WBGT (low-WBGT area, 20.79-25.15 °C; middle-WBGT area, 25.16-26.43 °C; and high-WBGT area, 26.44-29.26 °C).

**Table S9** Baseline characteristics of patients with one or more comorbidities (CCI ≥ 1)

|  | **Total** | **Low-WBGT**  **20.79-25.16 ℃** | **Middle-WBGT**  **25.17-26.43 ℃** | **High-WBGT**  **26.44-29.26 ℃** | **p-value** |
| --- | --- | --- | --- | --- | --- |
|  | **N=25,391** | **N=4,623** | **N=15,101** | **N=5,667** |  |
| **Personal-level variables** | |  |  |  |  |
| Age | 75.4(14.9) | 77.0(14.7) | 75.2(14.9) | 74.6(15.0) | <0.001 |
| Age category |  |  |  |  |  |
| <7 | 51(0.2) | 9(0.2) | 34(0.2) | 8(0.1) | <0.001 |
| 7-17 | 210(0.8) | 51(1.1) | 119(0.8) | 40(0.7) |  |
| 18-64 | 4,148(16.3) | 598(12.9) | 2,461(16.3) | 1,089(19.2) |  |
| 64-79 | 8,601(33.9) | 1,451(31.4) | 5,262(34.8) | 1,888(33.3) |  |
| 80 ≤ | 12,381(48.8) | 2,514(54.4) | 7,225(47.8) | 2,642(46.6) |  |
| Male | 15,421(60.7) | 2,642(57.1) | 9,251(61.3) | 3,528(62.3) | <0.001 |
| BMI category (kg/m^2^) |  |  |  |  |  |
| <18.5 | 4,911(19.3) | 871(18.8) | 3,011(19.9) | 1,029(18.2) | <0.001 |
| 18.5 – 24.9 | 14,705(57.9) | 2,726(59.0) | 8,688(57.5) | 3,291(58.1) |  |
| 25.0 – 29.9 | 4,301(16.9) | 787(17.0) | 2,498(16.5) | 1,016(17.9) |  |
| 30.0 ≤ | 1,096(4.3) | 191(4.1) | 644(4.3) | 261(4.6) |  |
| Missing | 378(1.5) | 48(1.0) | 260(1.7) | 70(1.2) |  |
| Smoking | 7,082(27.9) | 1,143(24.7) | 4,267(28.3) | 1,672(29.5) |  |
| Missing | 2,612(10.3) | 461(10.0) | 1,615(10.7) | 536(9.5) |  |
| Charlson Comorbidity Index | 1(1-2) | 1(1-2) | 1(1-2) | 1(1-2) | 0.015 |
| Myocardial infarction | 728(2.9) | 125(2.7) | 445(2.9) | 158(2.8) | 0.63 |
| Congestive  heart failure | 3,460(13.6) | 683(14.8) | 2,026(13.4) | 751(13.3) | 0.04 |
| Peripheral  vascular disease | 608(2.4) | 116(2.5) | 363(2.4) | 129(2.3) | 0.74 |
| Cerebrovascular disease | 4,902(19.3) | 917(19.8) | 2,853(18.9) | 1,132(20.0) | 0.13 |
| Dementia | 4,466(17.6) | 892(19.3) | 2,683(17.8) | 891(15.7) | <0.001 |
| Chronic  Pulmonary  disease | 2,163(8.5) | 376(8.1) | 1,322(8.8) | 465(8.2) | 0.26 |
| Rheumatic disease | 563(2.2) | 102(2.2) | 312(2.1) | 149(2.6) | 0.049 |
| Peptic ulcer disease | 1,442(5.7) | 322(7.0) | 876(5.8) | 244(4.3) | <0.001 |
| Mild liver disease | 2,712(10.7) | 411(8.9) | 1,571(10.4) | 730(12.9) | <0.001 |
| Diabetes without chronic complication | 6,909(27.2) | 1,207(26.1) | 4,104(27.2) | 1,598(28.2) | 0.06 |
| Diabetes with chronic complication | 1,668(6.6) | 280(6.1) | 1,024(6.8) | 364(6.4) | 0.19 |
| Hemiplegia/paraplegia | 141(0.6) | 27(0.6) | 86(0.6) | 28(0.5) | 0.78 |
| Renal disease | 1,737(6.8) | 293(6.3) | 1,028(6.8) | 416(7.3) | 0.13 |
| Malignancy | 2,625(10.3) | 546(11.8) | 1,562(10.3) | 517(9.1) | <0.001 |
| Moderate or severe liver disease | 158(0.6) | 21(0.5) | 96(0.6) | 41(0.7) | 0.21 |
| Metastatic solid tumor | 425(1.7) | 102(2.2) | 248(1.6) | 75(1.3) | 0.002 |
| AIDS/HIV | 9(0.0) | 0(0.0) | 9(0.1) | 0(0.0) | 0.047 |
| Mental disorder | 1,664(6.6) | 311(6.7) | 981(6.5) | 372(6.6) | 0.86 |
| **Admission year and month** | |  |  |  |  |
| Fiscal year |  |  |  |  | <0.001 |
| 2011 | 1,850(7.3) | 304(6.6) | 1,155(7.6) | 391(6.9) |  |
| 2012 | 1,668(6.6) | 354(7.7) | 957(6.3) | 357(6.3) |  |
| 2013 | 2,459(9.7) | 317(6.9) | 1,520(10.1) | 622(11.0) |  |
| 2014 | 1,824(7.2) | 378(8.2) | 1,065(7.1) | 381(6.7) |  |
| 2015 | 2,922(11.5) | 563(12.2) | 1,727(11.4) | 632(11.2) |  |
| 2016 | 2,997(11.8) | 492(10.6) | 1,646(10.9) | 859(15.2) |  |
| 2017 | 2,762(10.9) | 522(11.3) | 1,521(10.1) | 719(12.7) |  |
| 2018 | 5,247(20.7) | 877(19.0) | 3,322(22.0) | 1,048(18.5) |  |
| 2019 | 3,662(14.4) | 816(17.7) | 2,188(14.5) | 658(11.6) |  |
| Month |  |  |  |  | <0.001 |
| May | 734(2.9) | 163(3.5) | 384(2.5) | 187(3.3) |  |
| June | 1,424(5.6) | 251(5.4) | 855(5.7) | 318(5.6) |  |
| July | 11,057(43.5) | 1,854(40.1) | 6,709(44.4) | 2,494(44.0) |  |
| August | 10,991(43.3) | 2,150(46.5) | 6,485(42.9) | 2,356(41.6) |  |
| September | 1,185(4.7) | 205(4.4) | 668(4.4) | 312(5.5) |  |
| **Severity** |  |  |  |  |  |
| JCS category at admission |  |  |  |  | <0.001 |
| Alert | 16,706(65.8) | 3,002(64.9) | 9,867(65.3) | 3,837(67.7) | 0.021 |
| Dizzy | 6,195(24.4) | 1,161(25.1) | 3,719(24.6) | 1,315(23.2) |  |
| Drowsy | 1,461(5.8) | 282(6.1) | 872(5.8) | 307(5.4) |  |
| Come | 1,029(4.1) | 178(3.9) | 643(4.3) | 208(3.7) |  |
| ALF | 1,356(5.3) | 198(4.3) | 800(5.3) | 358(6.3) | <0.001 |
| AKI | 1,890(7.4) | 256(5.5) | 1,104(7.3) | 530(9.4) | <0.001 |
| DIC | 475(1.9) | 79(1.7) | 294(1.9) | 102(1.8) | 0.52 |
| **Intervention** |  |  |  |  |  |
| Intensive care unit | 1,058(4.2) | 103(2.2) | 748(5.0) | 207(3.7) | <0.001 |
| High care unit | 2,248(8.9) | 397(8.6) | 1,376(9.1) | 475(8.4) | 0.2 |
| Ambulance use | 16,476(64.9) | 2,818(61.0) | 10,034(66.4) | 3,624(63.9) | <0.001 |
| Catecholamine use | 889(3.5) | 153(3.3) | 542(3.6) | 194(3.4) | 0.62 |
| Mechanical  ventilation | 569(2.2) | 84(1.8) | 386(2.6) | 99(1.7) | <0.001 |
| Renal replacement therapy | 459(1.8) | 70(1.5) | 266(1.8) | 123(2.2) | 0.036 |
| **Outcomes** |  |  |  |  |  |
| In-hospital mortality | 715(2.8) | 175(3.8) | 404(2.7) | 136(2.4) | <0.001 |
| Length of hospital stay, median (IQR) | 7(4-17) | 7(4-17) | 8(4-17) | 7(4-16) | 0.005 |
| Hospitalization cost, median (IQR) | 2087(1172-4297) | 2041(1163-4331) | 2146(1203-4401) | 1972(1091-4015) | <0.001 |

Abbreviations; WBGT, wet bulb globe temperature; BMI, body mass index; CCI, Charlson Comorbidity Index; JCS, Japan Coma Scale; ALF, acute liver failure; AKI, acute kidney injury; DIC, disseminated intravascular coagulation; ICU, intensive care unit; HCU, high care unit; IQR, interquartile range; AIDS/HIV, acquired immunodeficiency syndrome/human immunodeficiency virus. Data are presented as numbers (%) unless otherwise indicated.

- Long-term local WBGT: The long-term average daily maximum WBGT for each prefecture during the study period.
- Local WBGT areas: We divided the 47 prefectures into three areas using the first and third quartiles of long term local WBGT (low-WBGT area, 20.79-25.15 °C; middle-WBGT area, 25.16-26.43 °C; and high-WBGT area, 26.44-29.26 °C).

**Table S10** Long-term average Wet-Bulb Globe Temperature (°C) across 47 prefectures.

| 01 | Hokkaido | 20.79 | 25 | Shiga | 25.97 |
| --- | --- | --- | --- | --- | --- |
| 02 | Aomori | 22.52 | 26 | Kyoto | 26.23 |
| 03 | Iwate | 23.17 | 27 | Osaka | 25.92 |
| 04 | Miyagi | 23.34 | 28 | Hyogo | 26.22 |
| 05 | Akita | 23.46 | 29 | Nara | 26.27 |
| 06 | Yamagata | 24.02 | 30 | Wakayama | 26.46 |
| 07 | Fukushima | 24.55 | 31 | Tottori | 26.17 |
| 08 | Ibaraki | 25.33 | 32 | Shimane | 25.86 |
| 09 | Tochigi | 25.16 | 33 | Okayama | 26.43 |
| 10 | Gunma | 25.24 | 34 | Hiroshima | 24.79 |
| 11 | Saitama | 26.52 | 35 | Yamaguchi | 23.71 |
| 12 | Chiba | 25.87 | 36 | Tokushima | 26.57 |
| 13 | Tokyo | 25.50 | 37 | Kagawa | 26.26 |
| 14 | Kanagawa | 26.07 | 38 | Ehime | 26.24 |
| 15 | Niigata | 24.03 | 39 | Kochi | 26.74 |
| 16 | Toyama | 25.42 | 40 | Fukuoka | 26.65 |
| 17 | Ishikawa | 25.42 | 41 | Saga | 26.65 |
| 18 | Fukui | 25.72 | 42 | Nagasaki | 26.72 |
| 19 | Yamanashi | 25.48 | 43 | Kumamoto | 26.97 |
| 20 | Nagano | 24.70 | 44 | Oita | 26.15 |
| 21 | Gifu | 26.28 | 45 | Miyazaki | 26.97 |
| 22 | Shizuoka | 26.01 | 46 | Kagoshima | 27.43 |
| 23 | Aichi | 25.90 | 47 | Okinawa | 29.26 |
| 24 | Mie | 25.96 |  |  |  |

Abbreviation; WBGT, wet bulb globe temperature.

**Table S11** Relative risks of covariates other than WBGT categories in the multivariable regression analysis predicting mortality

|  | **Adjusted odds ratio** | **95% CI** | **p value** |
| --- | --- | --- | --- |
| **Personal-level variables** |  |  |  |
| Age, per 1-year increase | 1.03 | (1.03-1.04) | <0.001 |
| Male | 1.03 | (0.93-1.13) | 0.538 |
| BMI category(kg/m^2^) |  |  |  |
| <18.5 | 1.92 | (1.70-2.17) | <0.001 |
| 18.5 - 24.9 | 1 (base) |  |  |
| 25.0 - 29.9 | 0.96 | (0.81-1.13) | 0.605 |
| 30.0 ≤ | 1.59 | (1.20-2.10) | 0.001 |
| Missing | 2.72 | (2.44-3.04) | <0.001 |
| Smoking | 1.26 | (1.22-1.31) | <0.001 |
| Charlson Comorbidity Index | 1.19 | (1.15-1.23) | <0.001 |
| Mental disorder | 0.87 | (0.71-1.06) | 0.152 |
| Transport by ambulance | 2.19 | (1.94-2.46) | <0.001 |
| **Fiscal year** |  |  |  |
| 2011 | 1 (base) |  |  |
| 2012 | 0.87 | (0.71-1.07) | 0.200 |
| 2013 | 0.74 | (0.61-0.90) | 0.003 |
| 2014 | 0.73 | (0.58-0.90) | 0.003 |
| 2015 | 0.69 | (0.57-0.84) | <0.001 |
| 2016 | 0.58 | (0.48-0.73) | <0.001 |
| 2017 | 0.60 | (0.49-0.73) | <0.001 |
| 2018 | 0.63 | (0.53-0.75) | <0.001 |
| 2019 | 0.76 | (0.64-0.92) | 0.004 |
| **Month** |  |  |  |
| May | 1 (base) |  |  |
| June | 1.27 | (0.88-1.84) | 0.200 |
| July | 1.70 | (1.24-2.34) | 0.001 |
| August | 1.53 | (1.11-2.10) | 0.010 |
| September | 1.09 | (0.74-1.62) | 0.663 |

Abbreviations; WBGT, wet bulb globe temperature; BMI, body mass index; CI, confidence interval.
